# Anti-MRSA Activity and Molecular Dynamics Simulations of a Novel Peptide from *Hevea brasiliensis* Latex Protein Waste Hydrolysates

**DOI:** 10.3390/life16101655

**Published:** 2026-10-01

**Authors:** Namfa Sermkaew, Apichart Atipairin, Sucheewin Krobthong, Chanat Aonbangkhen, Yodying Yingchutrakul, Jumpei Uchiyama, Nuttapon Songnaka

**Affiliations:** 1 School of Pharmacy, Walailak University, Thasala, Nakhon Si Thammarat 80160, Thailand; namfa.se@wu.ac.th (N.S.); apichart.at@wu.ac.th (A.A.); 2 Drug and Cosmetics Excellence Center, Walailak University, Thasala, Nakhon Si Thammarat 80160, Thailand; 3 Center of Excellence in Natural Products Chemistry (CENP), Department of Chemistry, Faculty of Science, Chulalongkorn University, Bangkok 10330, Thailand; sucheewin.k@chula.ac.th (S.K.); chanat.a@chula.ac.th (C.A.); 4 Center of Excellence on Petrochemical and Materials Technology, Chulalongkorn University, Pathumwan, Bangkok 10330, Thailand; 5 National Center for Genetic Engineering and Biotechnology, National Science and Technology Development Agency, Pathum Thani 12120, Thailand; yodying.yin@biotec.or.th; 6 Department of Infectious Diseases, Graduate School of Medicine, Dentistry and Pharmaceutical Sciences, Okayama University, Okayama 700-8558, Japan; uchiyama@okayama-u.ac.jp

**Keywords:** AMP, antimicrobial peptide, antimicrobial resistance, enzymatic hydrolysis, *Hevea brasiliensis*, molecular dynamics, MRSA, protease, rubber latex, *S. aureus*

## Abstract

The escalating methicillin-resistant *Staphylococcus aureus* (MRSA) crisis necessitates urgent therapeutic innovations. C-serum from *Hevea brasiliensis* is a protein-rich waste byproduct generated during rubber latex production. The primary objective of this research was to discover and evaluate a new antimicrobial peptide generated through the enzymatic digestion of C-serum protein waste. Enzymatic digestion liberated a novel cryptic hexapeptide, LLKKKY (CSPH-AMP). This peptide exhibited potent efficacy against planktonic and biofilm-associated *S. aureus* TISTR 517 and MRSA strains (142, 1096, and 2468). It demonstrated minimum inhibitory (MIC) and bactericidal (MBC) concentrations of 4 and 16 μg/mL, alongside minimum biofilm inhibitory (MBIC) and eradication (MBEC) concentrations of 16 and 128 μg/mL, respectively. SYTOX Green assays confirmed that the peptide induces rapid membrane permeabilization. Coarse-grained molecular dynamics simulations demonstrated that initial electrostatic peptide adsorption transitions into a bent transmembrane conformation, inducing localized lipid deformation and aqueous channel formation. Complementary proteomic analysis revealed that CSPH-AMP triggers targeted metabolic and structural cellular repair networks to counter membrane depolarization. Furthermore, CSPH-AMP demonstrated excellent thermal and proteolytic stability. By combining rapid membrane-lytic bactericidal action with robust physical stability, CSPH-AMP emerges as a promising, sustainably sourced candidate for future therapeutic development against drug-resistant MRSA.

## 1. Introduction

According to 2021 global mortality data, bacterial antimicrobial resistance (AMR) directly caused 4.7 million deaths. With 130,000 of these deaths attributed to methicillin-resistant *Staphylococcus aureus* (MRSA) infections, MRSA persists as a primary pathogen driving this crisis. Global forecasts suggest that by the year 2050, antimicrobial resistance may directly cause approximately 1.91 million deaths annually, while being indirectly linked to an additional 8.22 million fatalities worldwide [1]. Due to the escalating global incidence of MRSA, the development of innovative antimicrobial therapeutics to combat these resistant strains has become a critical priority.

Natural rubber latex is the specialized cytoplasm of laticifers found within the secondary phloem of the rubber tree, *Hevea brasiliensis*. This milky fluid, which contains a liquid serum, flows from the wound site when laticifers are breached, a biological feature exploited commercially to collect natural rubber. Numerous studies have documented the presence of antimicrobial proteins and peptides within the rubber tree. Specifically, several proteins and peptides found in the latex secretion, including Hev b5, hevein, hevamine, and 1,3-β-glucanase, as well as crude fractions derived from C-serum, exhibit a broad spectrum of antimicrobial activity against Gram-positive bacteria, Gram-negative bacteria, and fungi [2,3,4]. Natural rubber accounts for 20% to 60% of the total mass of fresh latex. When subjected to ultracentrifugation, the latex fractions into three distinct layers. The uppermost phase is composed of rubber particles, which are further classified by size into small and large groups; both variants feature a uniform spherical core encapsulated by a half-unit membrane. The intermediate aqueous phase, referred to as C-serum, harbors roughly 60% of the total protein content found in the latex. Finally, the dense bottom pellet is composed of lutoids, which are critical for coagulation processes and providing defense mechanisms against environmental pathogens [5,6].

The industrial processing of raw natural rubber produces large volumes of wastewater, which is released into the natural environment after treatment. The volume of wastewater generated is approximately 20 to 35 m^3^ per ton of collected natural rubber. This wastewater is highly polluted with nitrogen compounds and organic matter—particularly proteins, which can reach up to 2 kg per ton of fresh natural rubber [7]. Due to this high level of contamination, the effluent must be treated according to discharge standards before being released into water sources [7]. Proteomic investigations of rubber latex have identified nearly 200 proteins related to host defense systems and physiological homeostasis [8]. Given the high concentration of diverse proteins within these latex fractions, the residual wastewater generated during industrial processing serves as a significant, yet underutilized, source for the bioprospecting of bioactive compounds, most notably antimicrobial peptides.

Antimicrobial peptides (AMPs), typically consisting of 12 to 50 amino acids, were first recognized for their clinical potential in 1939 with the discovery of gramicidin from *Bacillus brevis*, which demonstrated efficacy against various Gram-positive bacteria [9]. Their amphipathic nature allows AMPs to physically penetrate and destabilize bacterial membranes through well-described biophysical mechanisms, including the carpet, toroidal pore, barrel-stave, and aggregate models. Because this fundamental mechanism targets general lipid bilayer architecture rather than specific, mutation-prone intracellular receptors, the AMP class is widely noted in the literature for possessing a higher barrier to resistance compared to traditional antibiotics [10]. Despite this distinct mechanistic advantage and their broad therapeutic potential, the clinical translation of AMPs faces several notable limitations. These primary challenges include a high susceptibility to proteolytic degradation, short in vivo half-lives, potential cytotoxicity against mammalian host cells [11]. While AMPs occur naturally, they can be produced in vitro via the enzymatic hydrolysis of various protein sources. Proteases are versatile across the industrial and biotechnological sectors, serving as foundational tools for both protein engineering and specialized research. These enzymes are essential for applications ranging from peptide synthesis to the removal of unwanted proteins during nucleic acid purification. Furthermore, their utility extends to cell culture maintenance, tissue dissociation, and the precise generation of recombinant antibody fragments for diagnostic and therapeutic purposes [12].

Driven by the dual imperatives of mitigating the global AMR crisis and advancing a sustainable bio-circular economy, this study aims to discover and characterize novel antimicrobial agents from industrial rubber latex waste. Specifically, enzymatic hydrolysis is utilized to liberate cryptic peptides from the protein-rich C-serum byproduct of *H. brasiliensis*. To comprehensively evaluate these peptides, this research integrates rigorous in vitro bactericidal assays with high-resolution in silico molecular dynamics (MD) simulations. By bridging macroscopic experimental data (such as potency, stability, and membrane-permeabilizing effects) with molecular-scale disruption, this multidisciplinary approach provides precise insights into the peptide–membrane disruption mechanism. Ultimately, this work highlights the immense strategic value of upcycling agricultural protein waste into vital biomedical innovations.

## 2. Materials and Methods

### 2.1. Protein Isolation from Natural Rubber Latex Production

This study focused on the valorization of discarded rubber proteins for the discovery of novel AMPs via protein hydrolysis [13]. C-serum was recovered from the natural rubber production process as wastewater (10 L) during the coagulation stage of sheet production from a *H. brasiliensis* plantation in Thung Song district, Nakhon Si Thammarat Province, southern Thailand (8°13′07.9” N 99°37′49.3” E). The collected samples were centrifuged at 14,500× *g* for 15 min. The resulting supernatant was filtered through a 0.22 μm cellulose nitrate membrane filter (Sigma-Aldrich, Warren, MI, USA) and subsequently subjected to heat-induced protein precipitation at 95 °C for 30 min. Subsequent to the heat treatment, the samples were subjected to centrifugation at 14,500× *g* for 15 min, and the resulting supernatant was decanted. To eliminate residual impurities, 10 g of the recovered protein pellet was washed three consecutive times using 30 mL of deionized water per wash. The purified pellet was then dehydrated in a hot air oven at 50 °C. The final desiccated product, designated as C-serum protein, was preserved at −80 °C for all downstream experimental applications.

### 2.2. Preparation of Protein Hydrolysates

Protein hydrolysates were produced by incubating 1 g of C-serum protein in 100 mL of 100 mM Tris–HCl buffer (pH 8.0) (AppliChem GmbH, Darmstadt, Germany) with various protease enzymes, including α-chymotrypsin (EC 3.4.21.1; ≥40 units/mg protein), proteinase K (EC 3.4.21.64; 30 mAnson-U/mg), and trypsin (EC 3.4.21.4; 1000–2000 BAEE units/mg) (Sigma-Aldrich, Warren, MI, USA) at a final enzyme concentration of 1 mg/mL [10]. Controls consisted of C-serum protein in 100 mM Tris–HCl buffer (pH 8.0) without enzymatic treatment. The reaction mixtures were incubated at 37 °C for 12 h, 24 h, and daily for 2–7 days. At the end of each incubation period, enzymatic hydrolysis was terminated by heat inactivation at 95 °C for 5 min. The samples were then centrifuged at 14,500× *g* for 15 min to separate the protein precipitate and protein hydrolysates present in the supernatant. The protein hydrolysates collected at each time point were used for subsequent antimicrobial assays.

### 2.3. Antimicrobial Activity Assay by Agar Well Diffusion Method

The antimicrobial efficacy of the crude protein hydrolysates in C-serum from *H. brasiliensis* was evaluated using the agar well diffusion method [14]. Representative pathogenic strains were obtained from the Thailand Institute of Scientific and Technological Research (TISTR) and the Medical Technology Laboratory at the Center for Scientific and Technological Equipment, Walailak University, Thailand. The tested isolates included *S. aureus* TISTR 517, MRSA strains 142, 1096, and 2468, *E. coli* TISTR 887, *K. pneumoniae* TISTR 1383, *P. aeruginosa* TISTR 357, *V. alginolyticus* TISTR 1572, *V. harveyi* TISTR 2088, and *V. parahaemolyticus* TISTR 1596. All strains were cultured in Mueller–Hinton broth (MHB) for 16–18 h to reach the exponential growth phase. Bacterial suspensions were standardized to an optical density (OD) of 0.1 at 625 nm (1 × 10^8^ CFU/mL) using sterile 0.85% (*w*/*v*) NaCl before spreading uniformly across the Mueller–Hinton (MH) agar plates. Protein hydrolysates were loaded from each digestion condition into the MHA wells at a volume of 100 µL per well. Following incubation at 37 °C for 16–18 h, the antimicrobial activity was determined by measuring the clear zones of inhibition. Measurements were performed manually utilizing a Vernier caliper. To ensure accuracy, two perpendicular diameters were measured across each well, and the resulting values were averaged. The standard antibiotics vancomycin and gentamicin were used as positive controls, whereas non-digested C-serum protein served as the negative control. All measurements were recorded in millimeters (mm) as the mean of triplicate experiments.

### 2.4. Purification of the AMP and Amino Acid Sequence Determination

The stepwise purification of the AMP from the crude protein hydrolysate was conducted using bioassay-guided fractionation [15]. The crude protein hydrolysate, obtained following a 72 h digestion with 1 mg/mL proteinase K, was assayed for antimicrobial activity prior to dialysis. The samples were dialyzed against deionized water for 16 h at 4 °C utilizing a 3.5 kDa molecular weight cut-off (MWCO) membrane (SnakeSkin, Pierce, Rockford, IL, USA). Following dialysis, the solution was evaporated to complete dryness using a vacuum concentrator (RVC 2-25 CDplus, Martin Christ, Osterode am Harz, Germany) to determine the total dry mass. Finally, the desiccated hydrolysate was reconstituted in Buffer A (50 mM ammonium acetate, 50 mM NaCl, pH 5.0) to achieve a concentration of 100 mg/mL. Partial purification was initiated via cation exchange (CIEX) chromatography. A 2 mL aliquot of the sample was applied to a 5 mL HiTrap SP column (GE Healthcare Bio-Sciences AB, Uppsala, Sweden) that had been equilibrated with Buffer A. Following a wash step with 25 mL of Buffer A (1 mL/min) to remove unbound materials, bound peptides were eluted applying a 50 mL linear gradient from 0% to 100% Buffer B (50 mM ammonium acetate, 1 M NaCl, pH 5.0). One-milliliter fractions were gathered and screened for anti-MRSA activity (strain 2468) using the agar well diffusion method. Fractions exhibiting antimicrobial efficacy were combined, dialyzed for desalting, and subsequently fractionated using a two-stage reversed-phase chromatography (RPC) protocol on an Inertsil ODS-3 C18 column (4.6 × 250 mm; GL Sciences, Tokyo, Japan). In the primary RPC step, 1 mL of the desalted pool was injected and eluted utilizing a 90 mL linear gradient from 0% to 100% Mobile Phase B (70% acetonitrile with 0.1% TFA) against Mobile Phase A (0.1% TFA in deionized water). The resulting 1 mL fractions were vacuum-dried, reconstituted in deionized water, and re-evaluated for anti-MRSA activity. To achieve high-resolution isolation of the target peptide, the active fraction from the first round was subsequently subjected to a second round of RPC utilizing a significantly shallower gradient. The same column and Mobile Phase A were employed; however, Mobile Phase B was substituted with a highly dilute formulation of 10% acetonitrile containing 0.1% TFA. The injected sample was eluted with a linear gradient of 0–100% of this dilute Mobile Phase B over a 90 mL volume. Fractions (1 mL) were again collected, evaporated, resuspended, and assayed for anti-MRSA activity. The highly purified active peak from this second round was finally selected for LC-MS/MS amino acid sequencing. The total dry weight of the active fractions from each purification step was recorded. The arbitrary activity (AU/mL) of the antimicrobial fractions was defined as the reciprocal of the highest two-fold dilution that still exhibited a visible zone of inhibition. The yield and enrichment of the entire purification process were summarized in a purification balance sheet.

To determine the primary amino acid sequence of the bioactive RPC fraction, LC-MS/MS analysis was performed utilizing an UltiMate 3000 liquid chromatograph coupled with a high-resolution mass spectrometer (Thermo Fisher Scientific, Waltham, MA, USA). Peptide separation was carried out on a reversed-phase C18 Hypersil Gold UHPLC column (4.6 × 30 mm; Thermo Fisher Scientific) at a flow rate of 300 µL/min. The mobile phase consisted of a 40-min linear gradient ranging from 0% to 100% acetonitrile supplemented with 0.1% formic acid. Mass spectrometric data were collected across an *m*/*z* range of 600 to 5000 in both positive and negative ionization modes. Operating parameters included a capillary temperature of 300 °C and a spray voltage of 3.2 kV, alongside a stepped normalized collision energy (NCE) protocol to facilitate comprehensive fragmentation of diverse peptide structures. The resulting spectra were analyzed for de novo sequencing via PEAKS Studio X software (version 13) (Bioinformatics Solutions Inc., Waterloo, ON, Canada) [16]. Subsequent sequence homology and physicochemical characterization were evaluated utilizing the Antimicrobial Peptide Database version 6 (APD6) [17], designating the characterized peptide as C-serum protein hydrolysate-derived AMP (CSPH-AMP). Candidate sequences were filtered based on mass spectrometry data quality, requiring an Average Local Confidence (ALC) score > 85% and a dominant relative abundance (peak area). Following analytical validation, the filtered sequences were evaluated against a non-redundant reference dataset of 954 antimicrobial peptides (AMPs). This dataset was compiled from a broader repository of 11,612 established AMPs sourced from the APD6, DBAASP, and DRAMP databases [17,18,19]. The physicochemical properties governing peptide–membrane interactions were computationally assessed. The net charge, hydrophobic percentage, and Grand Average of Hydropathicity (GRAVY) were calculated to evaluate amphipathicity. Furthermore, to predict interfacial membrane binding dynamics, the Wimley–White whole-residue hydrophobicity was assessed by estimating the Gibbs free energy of transfer (ΔG) from an aqueous environment to a neutral phosphatidylcholine (POPC) lipid bilayer interface. The candidate peptide was commercially synthesized (U2Bio Co., Ltd., Bangkok, Thailand) utilizing standard fluorenylmethyloxycarbonyl (Fmoc) solid-phase peptide synthesis (SPPS). Following sequential amino acid coupling and subsequent acidic cleavage from the solid resin support, the crude peptide was purified via reverse-phase high-performance liquid chromatography (RP-HPLC). The final peptide purity (≥95%) was determined using an analytical RP-HPLC system equipped with a YMC-Triart C18 column (250 × 4.6 mm, 3 µm particle size). A 30 µL aliquot of the sample was injected, and chromatographic separation was achieved using a binary solvent system comprising 0.1% trifluoroacetic acid (TFA) in water (Mobile Phase A) and 0.1% TFA in acetonitrile (Mobile Phase B). Peptide elution was continuously monitored at a wavelength of 214 nm with a constant flow rate of 1 mL/min. The gradient program was applied as follows: 0–20% B over 25 min, followed by an immediate increase to 100% B at 25.01 min, which was maintained until 30 min before system termination. Finally, accurate molecular mass and sequence integrity were verified by liquid chromatography–mass spectrometry (LC-MS) [20].

### 2.5. Determination of Planktonic and Biofilm Inhibitory Concentrations (MIC, MBC, MBIC, and MBEC) of CSPH-AMP

The antimicrobial and antibiofilm efficacies of both the purified C-serum hydrolysate-derived peptide fraction (natural CSPH-AMP) and the chemically synthesized candidate peptide were evaluated in parallel against *S. aureus* TISTR 517 and three MRSA strains (strains 142, 1096, and 2468). In accordance with CLSI guidelines [21], a planktonic broth microdilution assay was conducted utilizing cation-adjusted Mueller–Hinton broth (CAMHB). Bacterial inocula were prepared from 18 h MH agar precultures, suspended in sterile 0.85% NaCl, and adjusted to an optical density of 0.1 at 625 nm (approximately $1\times 10^{8}$ CFU/mL). This suspension was diluted 1:20 to yield a working inoculum of $5\times 10^{6}$ CFU/mL. Next, 10 μL aliquots of this suspension were inoculated into 96-well plates containing serial dilutions of either the natural or the synthesized peptide (ranging from 0.125 to 128 μg/mL), yielding a final assay volume of 100 μL and a bacterial density of $5\times 10^{5}$ CFU/mL per well. Vancomycin and cefoxitin were utilized as positive controls, while peptide-free wells served as negative growth controls. Following a 24 h incubation at 37 °C, the minimum inhibitory concentration (MIC) was defined as the lowest peptide concentration that completely suppressed visible planktonic growth. For the minimum bactericidal concentration (MBC) determination, 100 μL aliquots from the optically clear wells were subcultured onto MH agar. After an additional 24 h incubation, the lowest concentration resulting in zero colony growth was recorded as the MBC. Given that recalcitrant bacterial infections are frequently mediated by robust biofilms, the efficacies of both the natural and synthesized peptides were further assessed against initial biofilm formation as well as mature biofilms. The minimum biofilm inhibitory concentration (MBIC) was evaluated utilizing the 96-well plates from the initial MIC assay [22]. After 48 h of incubation, planktonic cells were carefully aspirated, and the wells were gently washed with phosphate-buffered saline (PBS) to eliminate non-adherent bacteria. The remaining adherent biomass was fixed and stained with 0.1% (*w*/*v*) crystal violet to quantify biofilm formation. The MBIC was designated as the lowest peptide concentration that effectively inhibited initial biofilm attachment relative to the untreated control. Furthermore, the minimum biofilm eradication concentration (MBEC) was determined to assess the capacity of the peptides to dismantle mature biofilms. Established biofilms were generated by incubating the standardized bacterial inoculum ($5\times 10^{5}$ CFU/mL) in 96-well plates at 37 °C for 48 h without antimicrobial exposure. The supernatant containing planktonic cells was subsequently discarded, and the established biofilms were gently washed with PBS. Fresh CAMHB containing serially diluted concentrations of the natural peptide, the synthesized peptide, vancomycin, or cefoxitin was then introduced to the wells. Following an additional 24 h incubation at 37 °C, the wells were washed and visually assessed. The MBEC was established as the lowest concentration required to completely eradicate the mature biofilm architecture. All planktonic and biofilm assays were conducted in independent triplicates to guarantee reproducibility, with the final reported MIC, MBC, MBIC, and MBEC values representing the statistical mode across the replicates.

### 2.6. Time-Kill Kinetics of CSPH-AMP

To investigate the bactericidal kinetics and mode of action of CSPH-AMP, time-kill assays were conducted against *S. aureus* TISTR 517 and three MRSA isolates (strains 142, 1096, and 2468). Bacterial suspensions in CAMHB were standardized to an initial density of $5\times 10^{5}$ CFU/mL. These cultures were subsequently treated with CSPH-AMP at final concentrations corresponding to 1×, 2×, 4×, and 8× the MIC. Cultures without peptide addition were maintained concurrently as untreated controls. All samples were incubated at 37 °C in 96-well microtiter plates [23]. At specific time intervals (0, 4, 8, 12, 16, 20, and 24 h), 100 μL aliquots were collected and spread onto MH agar plates. After a 24 h incubation period at 37 °C, the surviving colonies were counted. The kinetic data were graphically represented as log10 CFU/mL versus incubation time based on independent triplicate experiments. Statistical evaluations comparing the peptide-treated samples to the untreated controls at each time interval were executed using a two-way ANOVA coupled with Tukey’s post hoc test, with statistical significance defined at *p* < 0.05.

### 2.7. SYTOX Green Uptake Assay

The integrity of the cytoplasmic membrane following CSPH-AMP treatment was evaluated using a SYTOX Green uptake assay [24]. A 16 h culture was harvested, washed thrice with sterile PBS (pH 7.4) containing 0.2% CAMHB, and adjusted to an optical density (OD_625_) of 0.1 (1 × 10^8^ CFU/mL). Aliquots (100 μL) of this suspension were transferred to each well and pre-incubated with 2.5 μM SYTOX Green (Thermo Fisher Scientific Inc., Waltham, MA, USA) for 15 min in the dark. The cells were then treated with CSPH-AMP at concentrations of 0.5×, 1×, 2×, 4×, and 8× MIC. Fluorescence intensity was monitored for 24 h at excitation/emission wavelengths of 504/523 nm using a microplate reader (Tecan Group Ltd., Switzerland, Männedor). Statistical significance (*p* < 0.05) between treated and untreated groups was determined via two-way ANOVA followed by Tukey’s post hoc test.

### 2.8. Scanning Electron Microscopy (SEM) Analysis of AMP-Treated Bacterial Cells

Scanning electron microscopy (SEM) was utilized to observe the structural impact of CSPH-AMP on *S. aureus* TISTR 517 and MRSA strain 2468. Following an 18 h cultivation in MH broth, bacterial cells were centrifuged and washed thrice in sterile 0.85% NaCl to clear extracellular polysaccharides. The washed bacteria were resuspended in CAMHB, adjusted to an initial OD625 of 0.1, and diluted to yield $5\times 10^{5}$ CFU/mL. The cells were subsequently incubated for 16 h with CSPH-AMP or the positive control, vancomycin, both administered at 1× MIC. Sample preparation for SEM involved a 24 h fixation step in 0.1 M phosphate buffer (pH 7.2) containing 2.5% (*v*/*v*) glutaraldehyde. The samples were then dehydrated using a graded ethanol series and subjected to critical point drying (Quorum Technologies Ltd., Lewes, UK). Following gold sputtering, the cellular morphology was visualized at 20,000× magnification using a Carl Zeiss SEM (Oberkochen, Germany) [25].

### 2.9. Stability Assessment of CSPH-AMP

The stability of CSPH-AMP was assessed by quantifying its residual antimicrobial activity following exposure to diverse chemical and physical challenges [15]. To determine thermal stability, the peptide was incubated at 40, 60, 80 and 100 °C for up to 12 h, and its resilience to extreme sterilization conditions was tested via autoclaving at 121 °C (15 psi) for 15 and 30 min. The proteolytic susceptibility of the peptide was evaluated through incubation with a panel of enzymes at concentration of 1 mg/mL, including α-chymotrypsin (EC 3.4.21.1; ≥40 units/mg protein), proteinase K (EC 3.4.21.64; 30 mAnson-U/mg), and trypsin (EC 3.4.21.4; 1000–2000 BAEE units/mg). Furthermore, the peptide’s structural integrity was challenged across a broad pH spectrum (1.0, 6.8, and 7.4) and in the presence of 1% surfactants of varying ionic properties: sodium dodecyl sulfate (SDS, anionic), cetyltrimethylammonium bromide (CTAB, cationic), and Triton X-100 (non-ionic). Baseline activity was established using control samples stored at 4 °C. All post-treatment inhibitory effects were evaluated using an agar well diffusion assay against MRSA to determine whether the peptide retained functionality under these simulated industrial and physiological conditions.

### 2.10. Proteomics Analysis of CSPH-AMP Treatment Against Bacterial Pathogens

To assess the intracellular impact of CSPH-AMP, label-free quantitative proteomics was performed on *S. aureus* TISTR 517 and MRSA strain 2468. Bacteria were cultured with or without the peptide for 24 h in biological triplicates (*n* = 3) [26]. Following incubation, cell pellets were disrupted in lysis buffer (0.1× PBS at pH 7.4, 1% SDS, 5 mM DTT, and 1 mM PMSF) using a Sonics VCX-500 sonicator (Sonics & Materials, Inc., Newtown, CT, USA) (60% amplitude for 30 s). Lysates were clarified by centrifugation (10,000× *g*, 4 °C, 20 min), and proteins were subsequently precipitated with ice-cold acetone at −20 °C for 16 h. The resulting protein pellets were dissolved in 10 mM ammonium bicarbonate containing 0.25% RapiGest SF (Waters Corporation, Milford, MA, USA), and protein concentrations were determined via the Bradford assay. Aliquots of 25 µg of total protein were reduced (5 mM DTT at 90 °C for 15 min), alkylated in the dark at room temperature for 25 min, and desalted utilizing Zeba Spin columns (Thermo Fisher Scientific, Waltham, MA, USA). Proteins were then enzymatically cleaved with trypsin (1:50 *w*/*w*; Promega, Madison, WI, USA) at 37 °C for 6 h, and the generated peptides were reconstituted in 0.1% formic acid. LC-MS/MS analysis of 1.25 µg peptide samples was executed in positive ion mode using an EASY-nLC 1000 system interfaced with an Orbitrap HF mass spectrometer equipped with a nano-C18 column (Thermo Fisher Scientific, Waltham, MA, USA). Chromatographic separation utilized Mobile Phase A (0.1% formic acid in water) and Mobile Phase B (0.1% formic acid in 95% acetonitrile) through a sequential gradient: 2–45% B for 105 min, 90% B for 10 min, and 5% B for 35 min. Mass spectra were acquired in data-dependent acquisition (DDA) mode using higher-energy collisional dissociation (HCD) with a collision energy of 28. Full MS scans (*m*/*z* 400–1600) were obtained at a resolution of 120,000 and an AGC target of 3 × 10^6^ ions. Proteomic data processing and label-free quantification (LFQ) were conducted utilizing MaxQuant software (version 2.8.10) [27].

For protein annotation, the search parameters were configured with a mass error tolerance of 20 ppm and a false discovery rate (FDR) threshold of 0.01. Trypsin was specified as the digestion enzyme, with carbamidomethylation set as a fixed modification and methionine oxidation as a variable post-translational modification (PTM). LFQ was performed to identify expressed proteins by searching the dataset against the *Staphylococcus aureus* (strain NCTC 8325) UniProt reference proteome (Proteome ID: UP000008816; Taxon ID: 93061; containing 2889 protein sequences; accessed on 13 July 2026). This specific reference proteome was selected to provide a comprehensively annotated, standardized baseline for accurately mapping the pathogen’s expressed proteins and elucidating its biological response to CSPH-AMP treatment. To determine the biological pathways driving the pathogen’s response, the annotated protein lists were subjected to functional enrichment analysis. To ensure the reliability of the downstream functional and protein–protein interaction (PPI) analyses, the raw label-free quantification (LFQ) data was subjected to strict inclusion and exclusion criteria [28]. Proteins were excluded from the analysis if they were identified by fewer than two unique peptides or if they were not consistently detected in at least two out of three biological replicates. Proteins were classified as differentially expressed and included in the subsequent PPI network only if they met a statistical significance threshold of *p* < 0.05 and exhibited a relative fold-change of not less than 1.5 for upregulation or not more than 0.67 for downregulation compared to the untreated control group. The filtered list of differentially expressed proteins was then subjected to functional Gene Ontology (GO) analysis and comparative PPI network generation using the STRING database [29].

### 2.11. Proposed Membrane Interaction Mechanism of CSPH-AMP via Molecular Dynamics (MD) Simulations

To elucidate the interaction dynamics between CSPH-AMP and the bacterial envelope, molecular dynamics (MD) simulations were conducted using a representative *S. aureus* membrane model. The lipid bilayer was generated via the CHARMM-GUI Membrane Builder version 3.8, comprising a specific composition of 40% 1,2-O-dipalmitoyl-sn-glycero-3-phospho-(1′-rac-glycerol) (DPPG), 52% 1,2-O-dipalmitoyl-sn-glycero-3-phospho-rac-(3-lysyl(1′-glycerol)) (Lysyl-DPPG), and 8% 1,1′,2,2′-tetramyristoyl cardiolipin (TMCL). Following system assembly with Packmol version 21.0.1 [30], the peptide was initially positioned 50 Å above the membrane along the *z*-axis. The atomistic model was parameterized using the CHARMM36 all-atom force field [31]. The atomistic models of the peptide and membrane were converted into coarse-grained (CG) representations using the Martini force field. The CG system was solvated with the Martini water model, and Na^+^ and Cl^−^ ions were added to neutralize the system and achieve a final NaCl concentration of 0.15 M (150 mM) [32,33]. To ensure system stability prior to production runs, the system was subjected to energy minimization to eliminate steric clashes. During the initial equilibration phases, position restraints were applied to the peptide backbone and lipid headgroups to maintain structural integrity while the solvent relaxed. Following equilibration, production simulations were performed in the *NPT* ensemble at 310 K and 1 atm, utilizing a Berendsen thermostat and barostat (G & J Electronics Inc., Toronto, ON, Canada) with a time step of 20 fs. Trajectory analysis was conducted using custom Python scripts (version 3.9.0) and the MDAnalysis library (version 2.1) [34].

To fully characterize the proposed peptide–membrane interaction mechanism, several key parameters were calculated. For the peptide, molecular interactions were evaluated using various computational techniques. Specifically, MSD analysis was utilized to monitor the diffusive behavior of the peptide within the simulation environment, tracking its movement across the trajectory. Simultaneously, the MSD of the lipid bilayer was calculated to confirm the structural equilibration of the membrane both prior to and during peptide interaction [35]. RMSF analysis was used to determine how the peptide structure responded to the membrane environment, serving as a primary indicator of conformational changes over time [36]. The distance between the center of mass (COM) of the peptide and that of the membrane was a crucial determinant for evaluating how effectively the peptide penetrated the lipid bilayer and for identifying its most favorable location within the membrane [37]. The structural orientation of the peptide relative to the membrane was analyzed by measuring its tilt angle and internal bending angle during membrane interaction. The Gibbs free energy of peptide binding was evaluated by calculating the potential of mean force (PMF). The PMF was determined using the collective variables (Colvars) module and the adaptive biasing force (ABF) method, setting a fullSamples threshold of 500 and sampling along the *z*-axis across a 50-Å reaction coordinate relative to the membrane thickness [38]. Because spontaneous membrane insertion occurs on timescales that often exceed the kinetic sampling limits of conventional unbiased MD, ABF was employed to overcome these kinetic barriers. While ABF is an assisted sampling technique, it calculates the true thermodynamic landscape (Gibbs free energy) of the system. Therefore, identifying a free energy minimum along the reaction coordinate thermodynamically demonstrates that the partitioning of the peptide into the lipid bilayer is a spontaneous, energetically favorable process in real-time.

To investigate the specific peptide–membrane interactions, the contact frequency between individual peptide residues and the lipid bilayer was calculated across the trajectory. For this CG model, interacting residues were extracted using a standard distance cutoff of 6 Å. These interaction frequencies were then quantified to identify the key amino acids responsible for mediating peptide–membrane binding [15]. Membrane distortion induced by peptide insertion was quantified by calculating the radius of gyration (*R_g_*) of the lipid bilayer [39]. During peptide–membrane interaction, the perturbation induced by the peptide was evaluated using component density profiles. This analysis was combined with spatiotemporal visualization of the membrane surface to characterize pore formation and the subsequent leakage of water molecules as the bilayer lost its structural integrity [40,41]. Simulation trajectories of the CG peptide–membrane models were visualized via VMD 1.9.4. The simulations were executed in triplicate, utilizing a membrane system without the peptide as a control [42].

## 3. Results

### 3.1. Determination of Antimicrobial Potential of H. brasiliensis Latex Wastewater Protein Following Protease Hydrolysis

**Figure 1:**
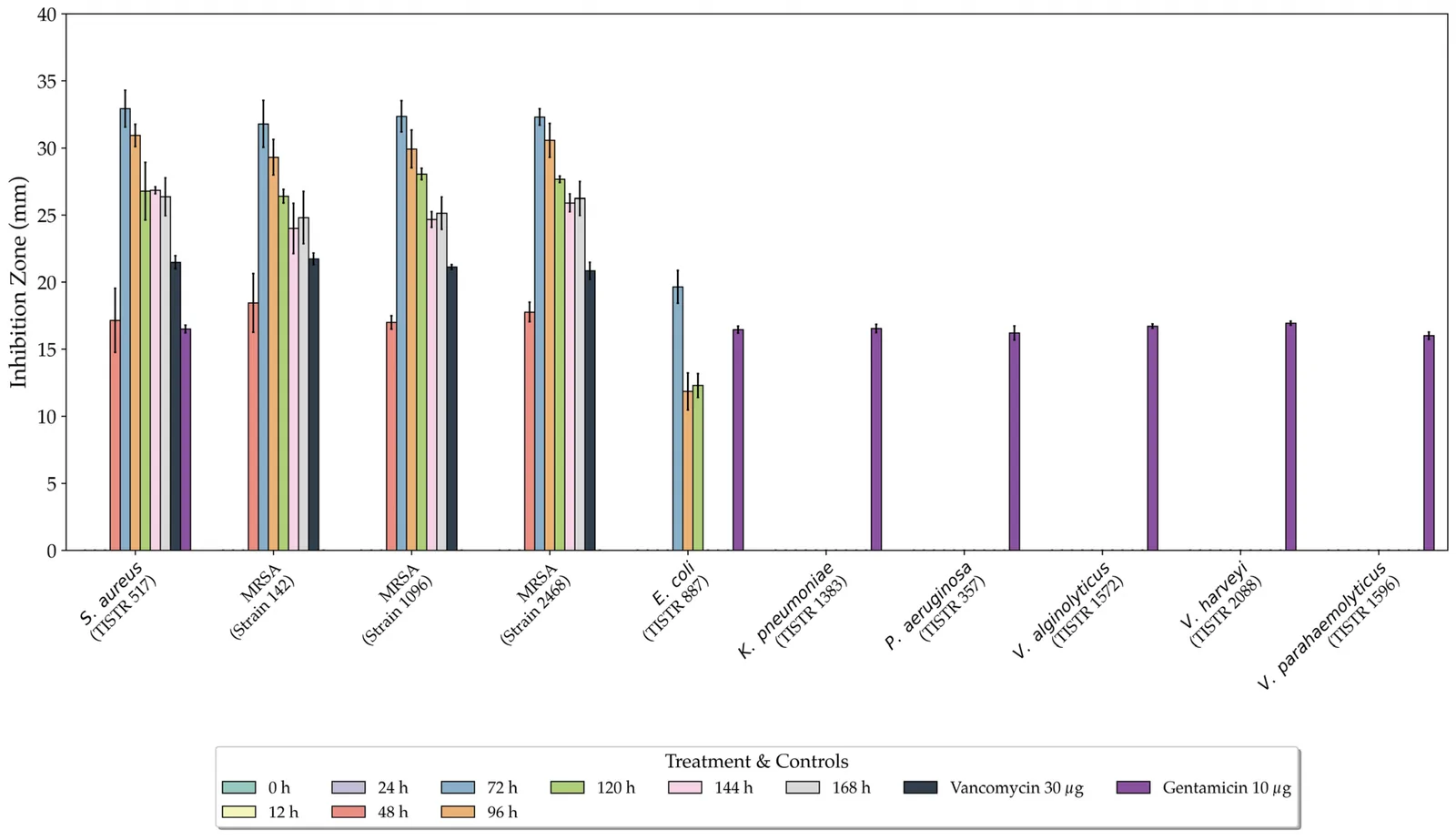
Antimicrobial activity of *H. brasiliensis* latex wastewater protein hydrolysates against selected bacterial pathogens, determined via agar well diffusion. The graph illustrates the time-dependent effect of proteinase K digestion on antimicrobial efficacy. Vancomycin (30 µg) and gentamicin (10 µg) served as standard positive controls. Values are presented as the mean inhibition zone diameter ± SD (*n* = 3) in millimeters.

The antimicrobial potential of *H. brasiliensis* latex wastewater protein was evaluated following enzymatic hydrolysis with three proteases: α-chymotrypsin, proteinase K, and trypsin. The findings indicated that the choice of protease significantly determined the bioactive profile and spectrum of the resulting protein hydrolysates. While α-chymotrypsin and trypsin yielded protein hydrolysates with narrow-spectrum activity that targeted only *S. aureus* TISTR 517 after prolonged incubation (96–120 h), proteinase K digestion yielded a diverse and potent antimicrobial profile. Notably, the proteinase K-derived hydrolysates demonstrated efficacy against *S. aureus* TISTR 517, three methicillin-resistant *Staphylococcus aureus* (MRSA) strains (strains 142, 1096, and 2468), and *E. coli* TISTR 887. Kinetic analysis revealed that proteinase K reached peak inhibitory activity at 72 h, significantly faster than the other tested enzymes. The resulting hydrolysates demonstrated a more pronounced potency against Gram-positive pathogens compared to Gram-negative strains. However, a decline in bioactivity was observed across all susceptible pathogens beyond the 72 h time point, likely due to secondary hydrolysis and the subsequent degradation of the active antimicrobial sequences into smaller, inactive fragments. Notably, the AMPs liberated by proteinase K yielded larger zones of inhibition against Gram-positive bacteria than the standard antibiotics vancomycin (30 μg) and gentamicin (10 μg). However, a definitive potency comparison between the liberated peptides and standard antibiotics can only be accurately established once the exact peptide concentration is determined following purification. None of the protein hydrolysates exhibited inhibitory activity against *K. pneumoniae* TISTR 1383, *P. aeruginosa* TISTR 357, or the tested *Vibrio* species (*V. alginolyticus* TISTR 1572, *V. harveyi* TISTR 2088, and *V. parahaemolyticus* TISTR 1596). Given its superior efficacy and broader inhibitory spectrum, proteinase K was identified as the most suitable biocatalyst for producing potent antibacterial peptides in this study (Figure 1). To maximize visual clarity, Figure 1 presents the time-dependent antimicrobial activity of the most potent proteinase K hydrolysates against susceptible strains. Graphical data corresponding to the narrowly active α-chymotrypsin and trypsin treatments, inactive time points, and non-susceptible bacterial strains have been omitted from the figure. Furthermore, the non-digested C-serum control exhibited no zones of inhibition, confirming that the observed antimicrobial activity is strictly dependent on enzymatic hydrolysis.

### 3.2. Purification and Structure Elucidation of the Antimicrobial Components of Crude Protein Hydrolysate in C-Serum

**Figure 2:**
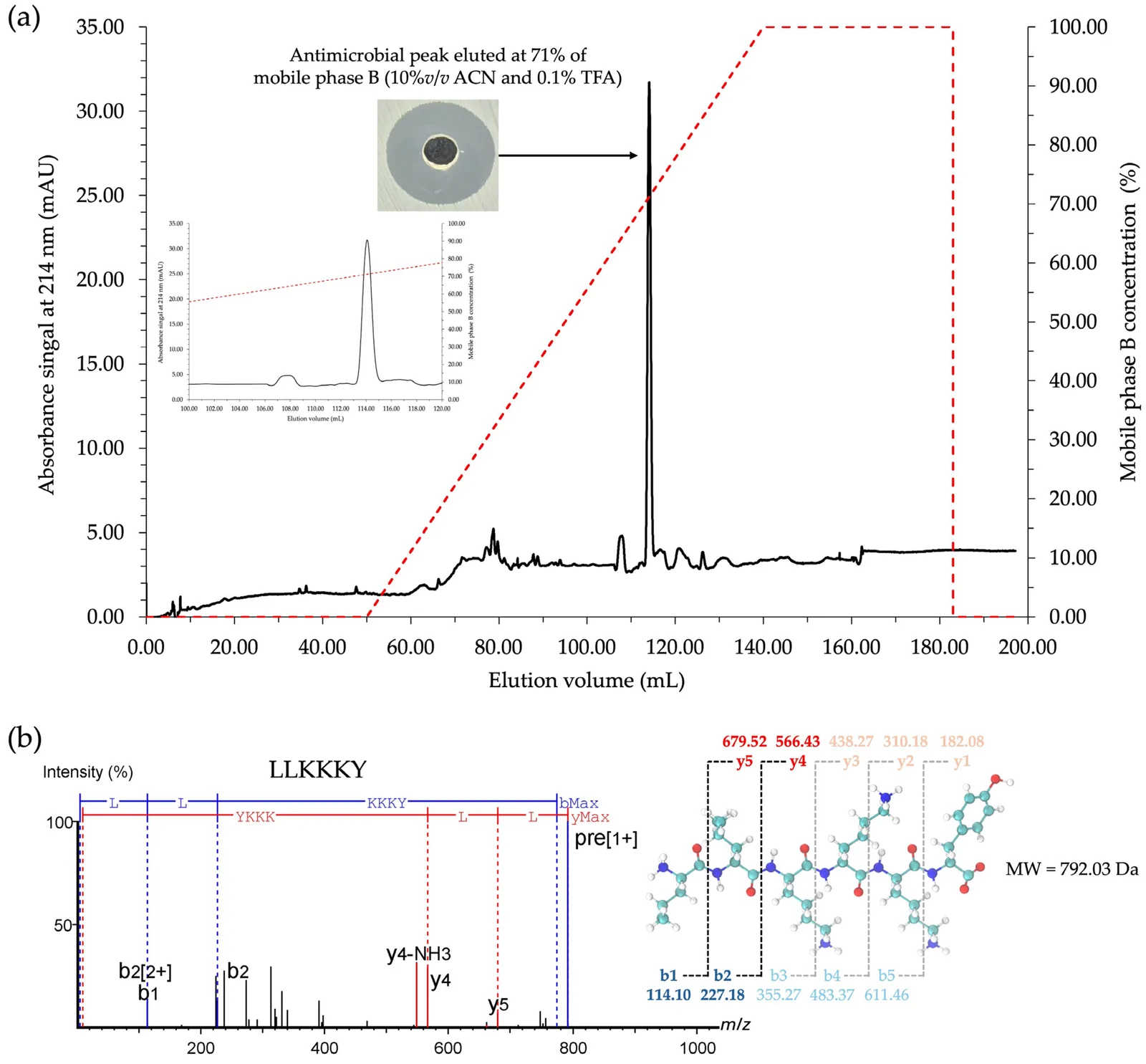
The purification of the CSPH-AMP was performed using reversed-phase chromatography. The indicated peak corresponds to the active fraction exhibiting antimicrobial activity against MRSA strain 2468, and the inset provides a magnified view of the eluted peak for clearer visualization. The solid black line represents the absorbance signal at 214 nm, and the red dashed line indicates the concentration gradient of mobile phase B (**a**). MS/MS spectrum for the struc-tural elucidation of the CSPH-AMP. Dark blue and red denote the experimentally observed b- and y-ion fragment mass-es, respectively, whereas pale blue and pale red represent the theoretical fragment masses. The dashed blue and red lines indicate the corresponding b-ion and y-ion cleavage sites along the peptide backbone. The final amino acid se-quence was determined using a de novo sequencing algorithm (**b**).

The crude protein hydrolysate derived from *H. brasiliensis* C-serum via proteinase K digestion was subjected to a multi-step purification sequence. As summarized in the purification profile (Table 1), a progressive reduction in total dry weight was observed across all stages, signifying the successful elimination of inactive components. Cation-exchange chromatography (CIEX) served as the initial fractionation step, yielding a specific activity of 169.92 AU/mg and a 21.57-fold purification factor. This active CIEX fraction was further resolved utilizing a two-step reversed-phase chromatography (RPC) strategy. During the first round of RPC, the active antimicrobial fraction eluted early in the gradient at 11.1% of Mobile Phase B (composed of 70% acetonitrile containing 0.1% trifluoroacetic acid [TFA]), which is equivalent to 7.77% absolute acetonitrile. To maximize purity, this collected fraction was subjected to a second round of high-resolution RPC employing a highly dilute organic gradient. The targeted active fraction eluted as a single major peak at 71% of this dilute Mobile Phase B (composed of 10% acetonitrile containing 0.1% TFA), which is equivalent to 7.10% absolute acetonitrile. Assays of the corresponding fractions confirmed that potent anti-MRSA activity was exclusively localized within this specific, highly hydrophilic peak, which was subsequently isolated for LC-MS/MS amino acid sequencing (Figure 2a). The final RPC stage resulted in a substantial increase in specific activity, reaching 970.06 AU/mg with a cumulative 123.17-fold purification. The process maintained a final yield of 7.56%, confirming the high efficiency of this chromatographic strategy in enriching the antimicrobial constituents from the complex C-serum matrix. Following the filtration of the active LC-MS/MS fraction, the C-serum protein hydrolysate-derived hexapeptide sequence LLKKKY (designated as CSPH-AMP) was selected as the primary candidate. This sequence demonstrated the highest peak area within the active fraction and exhibited an Average Local Confidence (ALC) score exceeding 85%, supporting the accuracy of the de novo prediction. The amino acid sequence of CSPH-AMP was supported by an average local confidence score of 87%, with a molecular weight of 792.03 Da (Figure 2b). Physicochemical analysis of CSPH-AMP indicated a net charge of +3, a grand average of hydropathicity (GRAVY) of −0.90, hydrophobicity of 0.91, and an instability index of −5.82, characterizing it as a stable, short, cationic peptide. The validation of the de novo sequencing results was performed to confirm that the observed biological efficacy was attributed to this specific sequence. The LLKKKY hexapeptide was chemically synthesized. The synthetic CSPH-AMP (achieving 99.33% purity and confirming the expected molecular mass of 792 Da) was subsequently evaluated for its planktonic and biofilm eradication profiles. Crucially, the synthetic peptide demonstrated MIC, MBC, MBIC, and MBEC values identical to those of the highly purified active fraction obtained from the final RPC stage. This direct functional parity definitively confirms that the LLKKKY sequence is the principal active antimicrobial constituent of the hydrolysate. Comparative bioinformatic analysis using the Antimicrobial Peptide Database (APD) indicated that CSPH-AMP shares 62.5% sequence homology with the de novo designed peptides L4K3W^4^ (LLKWLKKL; *S. aureus* MIC = 25 µg/mL) and L3K3W^4^ (LLKWLKK; *S. aureus* MIC = 200 µg/mL). Physicochemical profiling determined that CSPH-AMP has a GRAVY score of −0.90. Based on reference data from the Database of Antimicrobial Activity and Structure of Peptides (DBAASP), this value matched the GRAVY scores of peptide P9-4 (KWRRWIRWL), which has a GRAVY score of −1.31. Furthermore, the Wimley–White interfacial binding free energy for CSPH-AMP was calculated to be 0.91 kcal/mol. DRAMP database records indicate that this thermodynamic profile is comparable to the established heptapeptide KKKLAYA, which possesses an identical net charge (+3), a similar GRAVY score (−0.8), a Wimley–White value of 1.81 kcal/mol.

**Figure 3:**
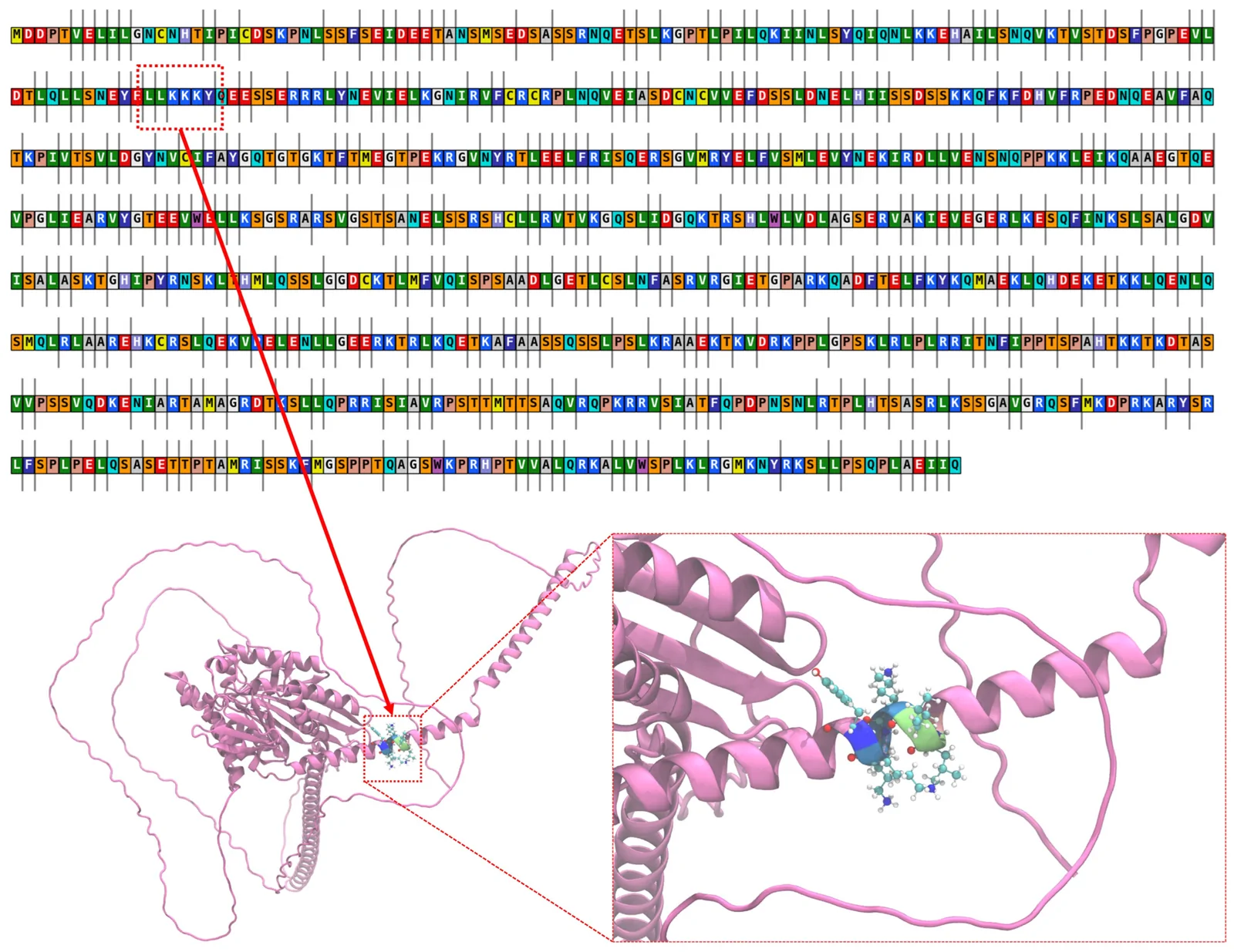
Primary amino acid sequence of the kinesin-like protein KIN-14S from *H. brasiliensis* rubber latex C-serum. The highlighted region indicates the location of the derived AMP, illustrating the specific alpha-helical fragment selectively released during proteinase K digestion, as confirmed by mass spectrometry.

The identification and sequence mapping of CSPH-AMP to its parent protein were performed via protein homology searching in the UniProt database (https://www.uniprot.org, accessed on 13 July 2026). The amino acid sequence of CSPH-AMP was queried against the UniProtKB sub-database, applying the BLOSUM62 substitution matrix and filtering specifically for *H. brasiliensis* proteins. The parent protein was identified as kinesin-like protein KIN-14S (XP_021653015.2), a known constituent of rubber latex C-serum [43]. Within this parent protein, the exact “LLKKKY” sequence of CSPH-AMP is flanked by a phenylalanine residue at its N-terminus and a tyrosine residue at its C-terminus. These aromatic amino acids are highly susceptible to proteinase K digestion. This finding confirms that proteinase K proteolysis is an effective strategy for liberating short, cryptic AMPs from larger precursor proteins (Figure 3). Interestingly, searching the CSPH-AMP amino acid sequence against major AMP databases, including APD6, DRAMP, and DBAASP, yielded no exact matches to any known AMPs, thereby confirming the novelty of this specific sequence. Furthermore, within the native parent protein, this peptide segment exhibits an alpha-helical structure, which was subsequently adopted as the initial conformation for the MD simulations.

### 3.3. Determination of Antibacterial and Antibiofilm Activity of CSPH-AMP by Microdilution Assay

The antimicrobial and antibiofilm potencies of both the naturally purified CSPH-AMP fraction and the chemically synthesized candidate peptide were determined using a microdilution assay and compared against the clinical standards, vancomycin and cefoxitin (Table 2). Both the natural and synthesized peptides exhibited identical bioactivity profiles across all assays. For both *S. aureus* TISTR 517 and the MRSA strains, the MBC of the natural and synthesized CSPH-AMP (16 μg/mL) was 4-fold higher than its MIC (4 μg/mL). Although vancomycin maintained superior absolute potency with MIC and MBC values of 2 μg/mL across all strains, the therapeutic relevance of CSPH-AMP was highlighted by its consistent efficacy against MRSA; in contrast, these strains displayed high-level resistance to cefoxitin (MIC and MBC > 128 μg/mL). The antibiofilm efficacy of the peptides was further evaluated by determining the MBIC and MBEC. For both forms of CSPH-AMP, the MBIC and MBEC values were 4-fold (16 μg/mL) and 32-fold (128 μg/mL) higher than the MIC, respectively, against all tested *S. aureus* and MRSA strains. While vancomycin achieved antibiofilm effects at lower absolute concentrations than CSPH-AMP, it exhibited an identical proportional increase, requiring 4-fold and 32-fold its MIC to achieve the MBIC (8 μg/mL) and MBEC (64 μg/mL), respectively. Conversely, cefoxitin successfully inhibited and eradicated biofilms of the methicillin-susceptible *S. aureus* TISTR 517 strain at 2-fold (4 μg/mL) and 16-fold (32 μg/mL) its MIC, but completely failed to exhibit antibiofilm activity against any MRSA strains at the maximum tested concentration (>128 μg/mL).

### 3.4. Time-Kill Kinetics of CSPH-AMP Against S. aureus and MRSA

**Figure 4:**
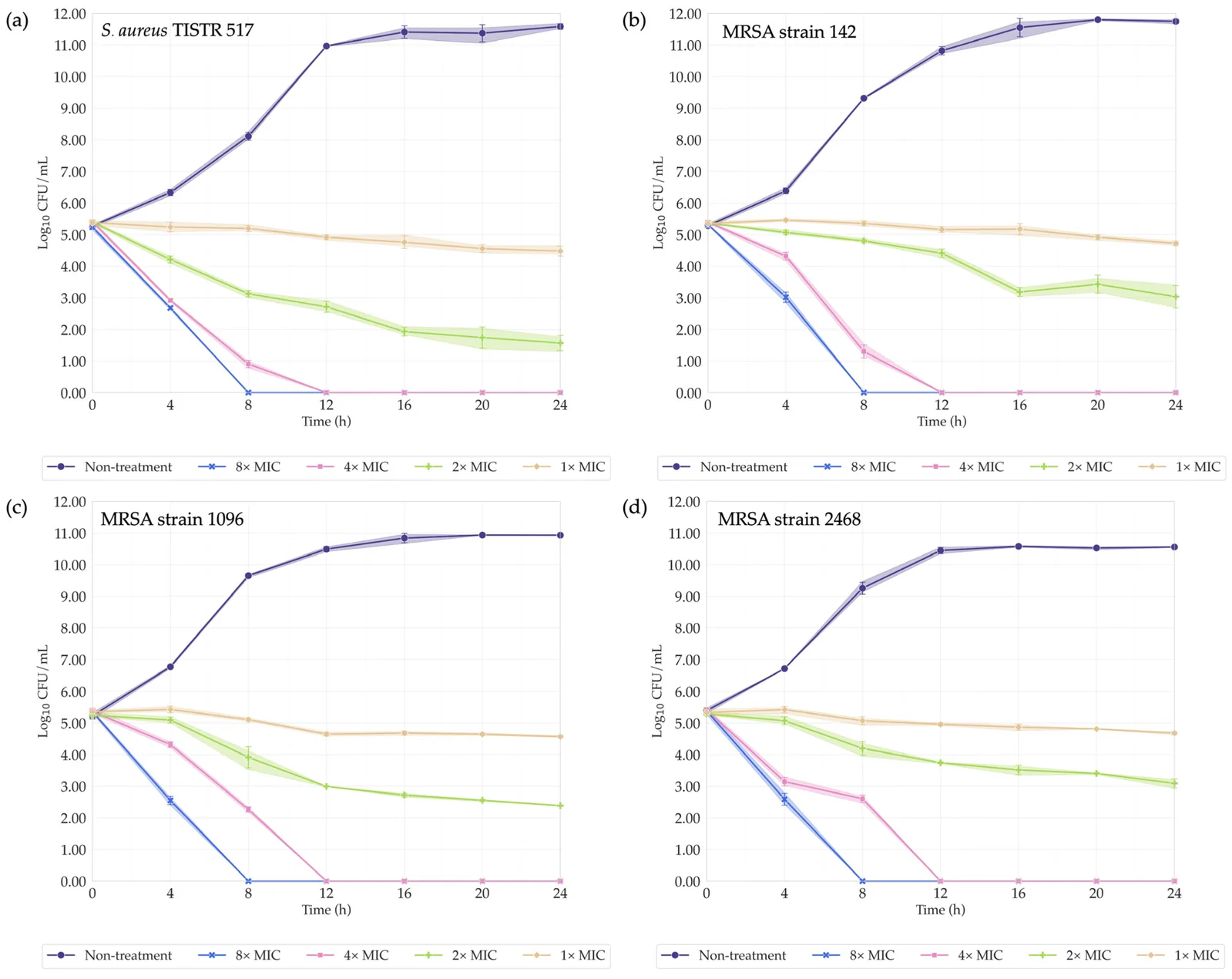
Time-kill curves of *S. aureus* TISTR 517 (**a**) and MRSA strain 142 (**b**), MRSA stain 1096 (**c**), and MRSA strain 2468 (**d**) treated with various concentrations of CSPH-AMP. Bacterial viability was quantified as log CFU/mL over a 24 h period following exposure to 1× MIC (t), 2× MIC (+), 4× MIC (n), and 8× MIC (×). The non-treatment (l) was included for baseline comparison. Data points represent the mean ± SD (*n* = 3), and the shaded areas indicate the 95% confidence intervals.

The time-kill kinetics of CSPH-AMP were evaluated against *S. aureus* TISTR 517 and MRSA strains 142, 1096, and 2468 at concentrations of 1×, 2×, 4×, and 8× the MIC (Figure 4a–d). In the absence of treatment, significant bacterial growth was observed across all strains. Conversely, treatment with the AMP resulted in a concentration-dependent decrease in bacterial viability. Notably, from 4 h of treatment onward, all tested peptide concentrations demonstrated a statistically significant reduction in viable cells (*p* < 0.05) when compared to the non-treatment control condition. At 1× MIC, the log reduction rates for *S. aureus* TISTR 517 and MRSA strains 142, 1096, and 2468 were −0.040, −0.028, −0.039, and −0.030 log CFU/h, respectively. When the concentration was increased to 2× MIC, the rates shifted to −0.157, −0.106, −0.133, and −0.095 log CFU/h. Notably, at lower concentrations (1× and 2× MIC), *S. aureus* TISTR 517 was eradicated more rapidly than the MRSA strains. At higher concentrations, the efficacy became more uniform across all tested bacteria. At 4× MIC, the log reduction rates for *S. aureus* TISTR 517 and the three MRSA strains were −0.456, −0.480, −0.454, and −0.412 log CFU/h. At 8× MIC, the rates reached −0.654, −0.665, −0.670, and −0.674 log CFU/h, respectively. At these elevated concentrations (4× and 8× MIC), the killing rates of *S. aureus* TISTR 517 and the MRSA strains were comparable. Complete eradication was achieved at 4× and 8× MIC, with no viable bacteria detected at 12 h and 8 h, respectively, for all strains. Overall, these time-kill kinetics demonstrate that the bactericidal activity of CSPH-AMP is strongly concentration-dependent.

### 3.5. Bacterial Membrane Permeabilization Assay

**Figure 5:**
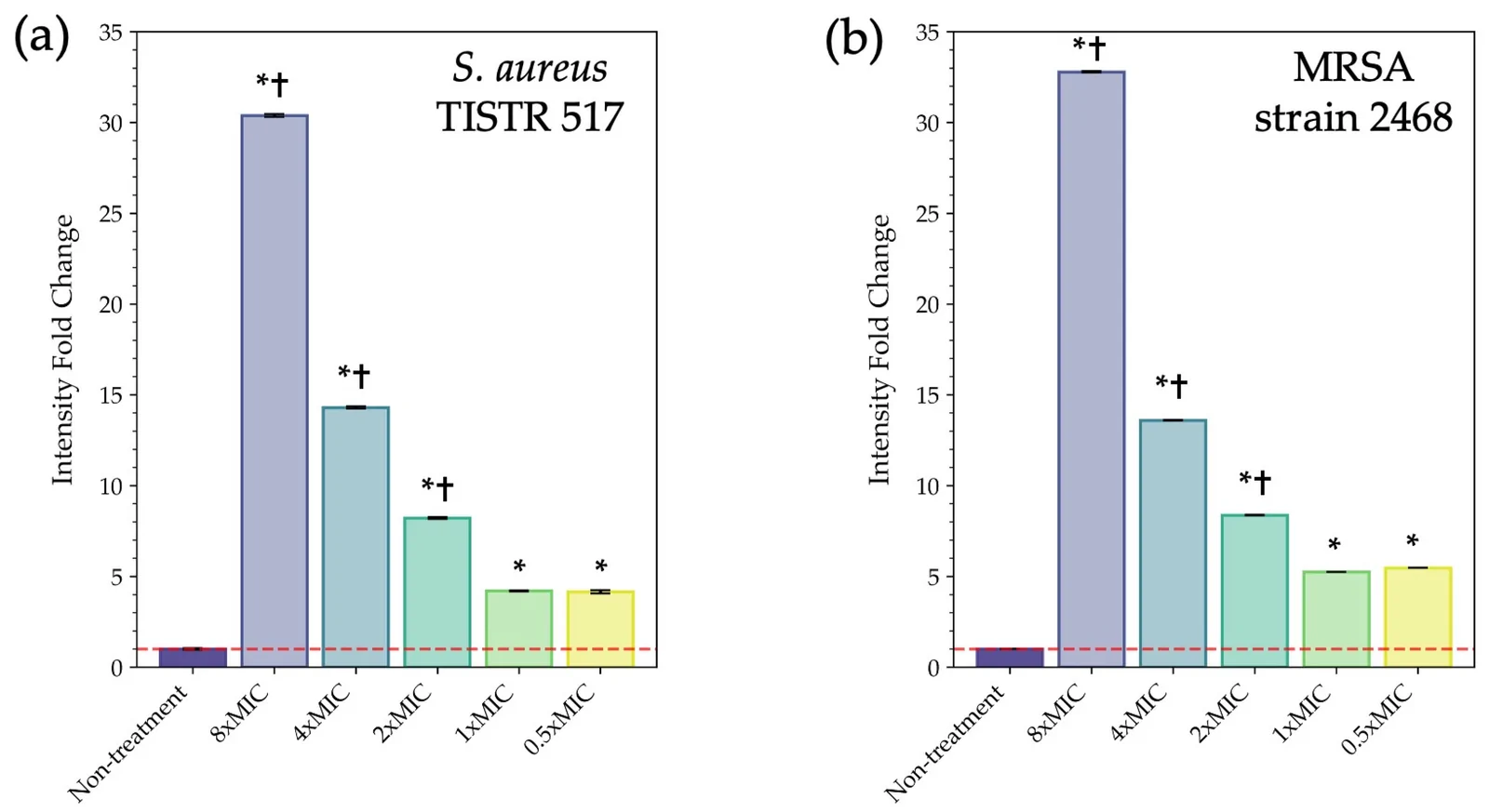
Quantitative assessment of bacterial membrane permeabilization induced by CSPH-AMP, measured via SYTOX Green fluorescence. The histograms illustrate the fold change in fluorescence intensity relative to the untreated control for (**a**) S. aureus TISTR 517 and (**b**) MRSA strain 2468 following treatment with CSPH-AMP at concentrations ranging from 0.5× to 8× MIC. Data represent the mean of triplicate independent experiments. The horizontal red dashed line represents the baseline fluorescence level of the untreated control (fold change = 1). Statistical significance (*p* < 0.05) is denoted by an asterisk (*) for comparisons between treatments and the non-treated control, and by a dagger (†) for comparisons between a specific treatment concentration and its adjacent lower concentration.

To evaluate the bacterial membrane permeabilization potential of CSPH-AMP, a SYTOX Green uptake assay was performed against *S. aureus* TISTR 517 and MRSA strain 2468 (Figure 5a and Figure 5b, respectively). Initial fluorescence was monitored for 5 min prior to peptide addition to confirm baseline cell integrity. Following the introduction of the AMP at concentrations of 0.5×, 1×, 2×, 4×, and 8× MIC, an immediate increase in fluorescence intensity was observed. After this initial sharp spike, the fluorescence rapidly reached a steady state. These kinetics indicate that the peptide molecules spontaneously insert into and destabilize the bacterial membrane. Furthermore, at concentrations ranging from 1× to 8× MIC, CSPH-AMP induced a statistically significant (*p* < 0.05), concentration-dependent increase in fluorescence. This increase signifies the formation of SYTOX Green-DNA complexes as a direct result of membrane compromise. At the highest tested concentration (8× MIC), the AMP induced a substantial 30-fold and 33-fold increase in fluorescence for *S. aureus* TISTR 517 and MRSA, respectively, compared to the untreated controls. The degree of permeabilization remained comparable between both strains at lower concentrations (of 0.5×, 1×, 2×, and 4× MIC). Notably, the fluorescence intensities observed at 0.5× MIC indicate that the bacterial membranes of both *S. aureus* TISTR 517 and MRSA strain 2468 were compromised even at sub-inhibitory concentrations.

### 3.6. Scanning Electron Microscopy (SEM) Analysis of Bacterial Morphological Changes Following AMP Treatment

**Figure 6:**
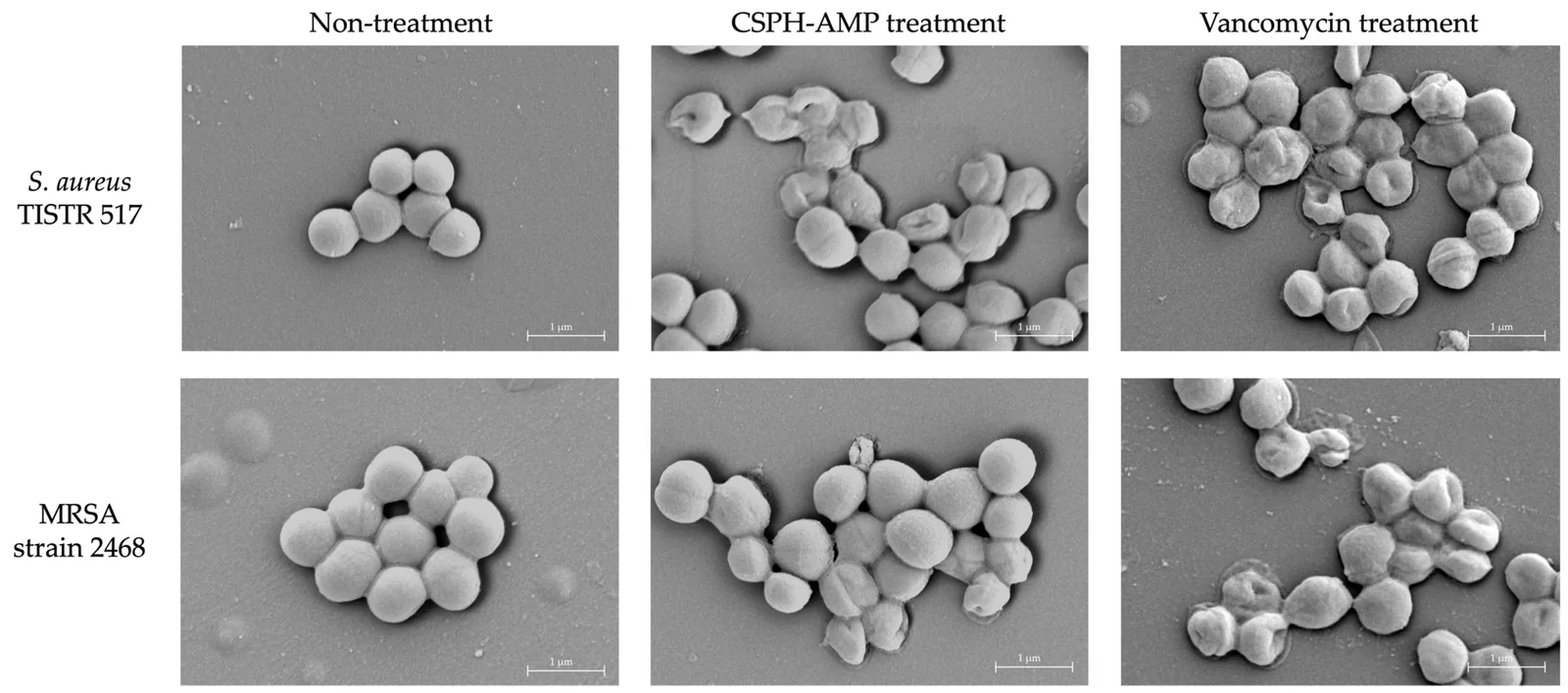
Comparative ultrastructural analysis of *S. aureus* TISTR 517 and MRSA strain 2468 via SEM. The effects of CSPH-AMP on bacterial morphology are compared against non-treatment condition and a vancomycin-treated positive control.

The ultrastructural changes in *S. aureus* TISTR 517 and MRSA strain 2468 upon treatment with CSPH-AMP were investigated using SEM. In the untreated control groups, both *S. aureus* and MRSA cells exhibited intact, spherical morphologies with characteristically smooth surfaces. Following treatment with CSPH-AMP at a concentration of 1× MIC for 16 h, significant morphological distortion was observed in *S. aureus* TISTR 517 and MRSA strain 2468, characterized by cell shrinkage and a loss of the typical spherical structure. Notably, localized indentations were clearly visible on the bacterial membranes. The structural alterations induced by CSPH-AMP were comparable to those observed in the vancomycin-treated group, which served as a positive control. Overall, these morphological changes demonstrated clear membrane disruption in both the methicillin-susceptible and methicillin-resistant strains (Figure 6).

### 3.7. Stability Studies of CSPH-AMP

The stability of the purified active fraction containing CSPH-AMP, obtained from the final reversed-phase chromatography (RPC) stage. The stability of CSPH-AMP was evaluated under various environmental challenges to assess its physicochemical robustness (Table 3). The thermal resilience of CSPH-AMP was investigated across a range of temperatures (40–100 °C) and under autoclave conditions. CSPH-AMP maintained robust stability at temperatures up to 100 °C, showing no significant loss in anti-MRSA activity compared to the control stored at 4 °C. However, exposure to autoclave conditions (121 °C, 15 psi) resulted in a significant, time-dependent reduction in residual activity after 15 and 30 min of treatment. This suggests that the peptide is highly thermostable, maintaining its activity following a 12 h treatment at temperatures up to 100 °C, although it is slightly susceptible to degradation under the extreme combined stress of high temperature (121 °C), high pressure, and saturated steam during autoclaving.

The protease susceptibility of CSPH-AMP was also monitored following incubation with various exogenous proteases, including proteinase K, trypsin, and α-chymotrypsin. The peptide remained stable during the initial 1 h of enzymatic exposure. A time-dependent decrease in antimicrobial potency was observed thereafter, extending up to 12 h of treatment, with approximately 85% of the initial activity remaining. This observed reduction in antimicrobial activity directly aligns with the identified primary sequence of CSPH-AMP (LLKKKY) and the specific cleavage preferences of the tested enzymes. The sequence contains a core motif of three consecutive lysine (K) residues, rendering it highly susceptible to targeted cleavage by trypsin. Concurrently, the terminal leucine (L) and tyrosine (Y) residues provide ideal cleavage sites for α-chymotrypsin and proteinase K. Consequently, prolonged enzymatic exposure progressively degrades these specific structural motifs, leading to the decline in activity observed at 6 and 12 h. To evaluate formulation compatibility, the interaction of CSPH-AMP with various surfactants was evaluated. Incubation with the cationic surfactant CTAB did not alter the peptide’s anti-MRSA activity over 12 h, with results comparable to CTAB alone. The addition of SDS and Triton X-100 appeared to enhance the overall antimicrobial effect. Notably, the combination of CSPH-AMP and Triton X-100 yielded the highest observed activity among all experimental groups. Given that Triton X-100 alone exhibited no activity against MRSA, these results strongly suggest a synergistic interaction between the peptide and the non-ionic detergent in disrupting the MRSA membrane.

The stability of CSPH-AMP was further investigated across a physiological pH range (pH 1.2 to 7.4). While the peptide maintained stable activity at near-neutral conditions (pH 6.8 and 7.4), a substantial enhancement in antimicrobial performance was observed under acidic conditions (pH 1.2 and 4.5). This suggests that the protonation of specific amino acid residues at low pH may increase the net positive charge of the peptide, thereby improving its electrostatic attraction to the bacterial surface.

### 3.8. Comparative Protein–Protein Interaction (PPI) Network Topology and Functional Gene Ontology (GO) Clustering of S. aureus TISTR 517 and MRSA Strain 2468 in Response to CSPH-AMP

**Figure 7:**
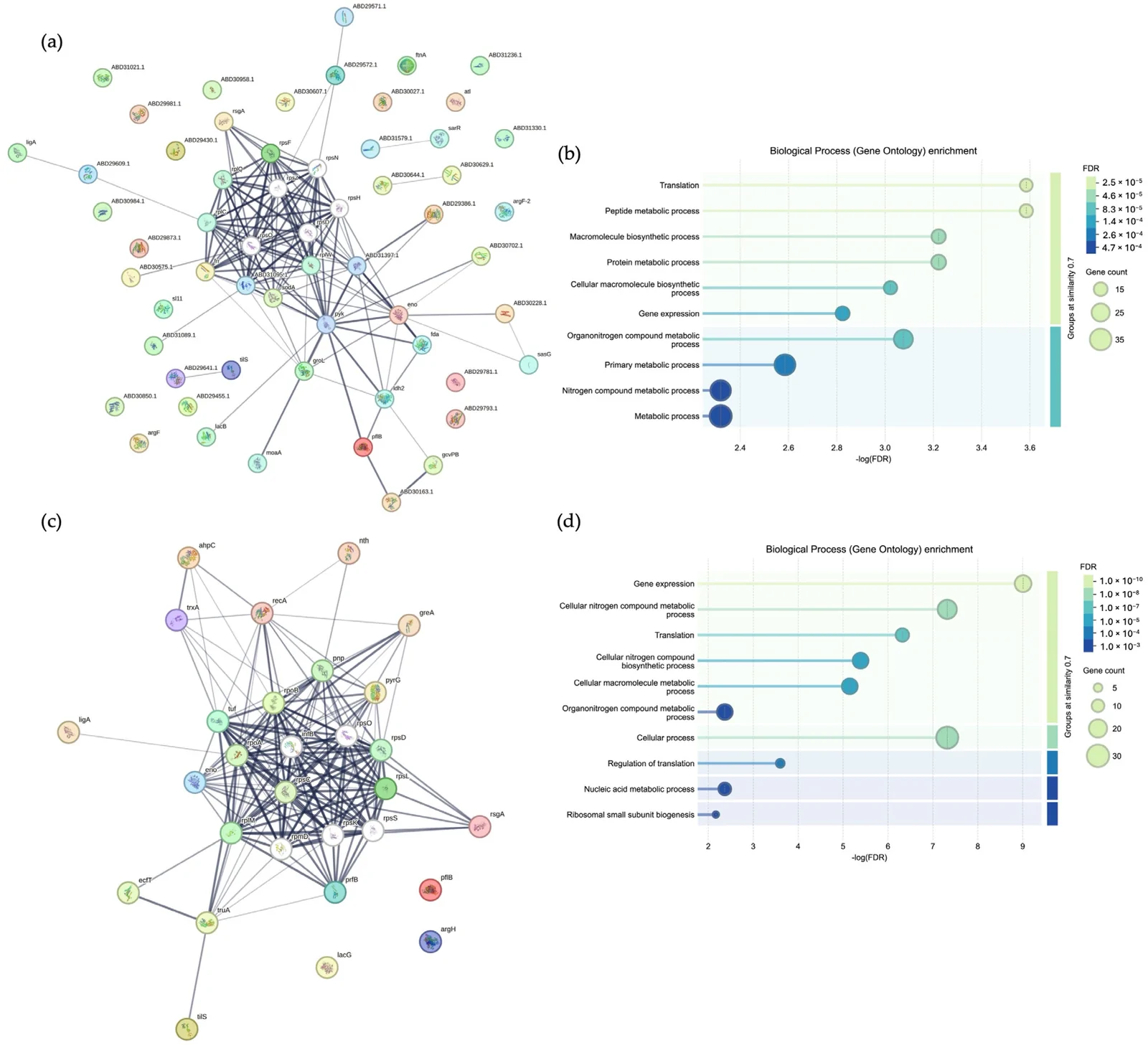
Comparative protein–protein interaction (PPI) networks and functional Gene Ontology (GO) enrichment analysis of *S. aureus* TISTR 517 and MRSA strain 2468 following CSPH-AMP treatment. PPI networks for *S. aureus* TISTR 517 (**a**) and MRSA strain 2468 (**c**) were generated via the STRING database. Bubble plots represent enriched Biological Process (BP) Gene Ontology terms for *S. aureus* TISTR 517 (**b**) and MRSA strain 2468 (**d**), respectively. The *x*-axis indicates the −log_10_ (FDR), where values > 1.3 are considered statistically significant. Bubble size corresponds to the gene count (number of proteins associated with the term), while colors represent functional clusters identified through a similarity index threshold of 0.7.

The functional landscape of the proteome in *S. aureus* TISTR 517 and MRSA strain 2468 following CSPH-AMP treatment was visualized using STRING-generated PPI networks (Figure 7). Under CSPH-AMP treatment, a total of 50 and 41 proteins were significantly upregulated in *S. aureus* TISTR 517 and MRSA strain 2468, respectively (Table S1).

In *S. aureus* TISTR 517, the upregulated proteome was primarily centered on alternative metabolic pathways and stress-survival mechanisms. Notable proteins included molybdopterin cofactor biosynthesis protein A (*moaA*), suggesting a metabolic shift toward anaerobic fermentation to compensate for energy deficits caused by membrane depolarization. Furthermore, the upregulation of ornithine carbamoyltransferase (*argF-2*) indicated the activation of arginine metabolism, a known strategy for maintaining pH homeostasis against the ionic imbalances induced by the AMP. The defensive response was further evidenced by the increased production of superoxide dismutase [Mn] 1 (*sodA*) and ABD31330.1, confirming that CSPH-AMP induced significant oxidative stress, prompting the bacteria to neutralize reactive oxygen species (ROS) and prevent DNA-protein cross-linking (Figure 7a).

In MRSA strain 2468, the response was characterized by the upregulation of diverse biological transporters and structural repair enzymes. Transmembrane proteins, including the FecCD family (*SAR2266*), ABC-2 type transport systems (*SAR2595*), and the cobalt transporter (*ecfT*), were significantly enriched, likely acting as efflux pumps to expel the peptide or restore metal ion homeostasis. Additionally, the upregulation of Penicillin-binding protein PBP2B (*pbpF*), which functions in peptidoglycan biosynthesis, and DNA ligase (*ligA*) underscores a coordinated effort to repair cell wall damage and maintain genomic integrity in response to the AMP. Other critical responses included lipid metabolism (*ugpQ*) and probable cytosol aminopeptidase (*pepA*), reflecting a broad-scale reorganization of the cellular primary metabolism (Figure 7c).

Functional impact assessment of differentially expressed proteins in *S. aureus* TISTR 517 and MRSA strain 2468 following CSPH-AMP treatment was conducted through the STRING database. Statistical significance was defined by a threshold of False Discovery Rate (FDR) < 0.05 [44]. To minimize biological redundancy, Gene Ontology (GO) terms were clustered using a high functional similarity index of 0.7. The convergence of high gene counts and significant FDR values across these clusters provided robust evidence that CSPH-AMP targets the core structural and metabolic integrity of both pathogens. The gene count, represented by bubble size, reflects the total number of proteins associated with each GO term. Higher values on this scale indicate a greater level of confidence in the observed enrichment. In *S. aureus* TISTR 517, pathways related to translation and peptide metabolic processes exhibited the highest statistical significance (Figure 7b and Table S2), suggesting that CSPH-AMP treatment compromises protein synthesis and gene expression, thereby triggering a cell survival response. Similarly, gene expression pathways emerged as the most significantly enriched category in MRSA strain 2468. However, the magnitude of this expression was higher in the resistant strain, reflecting the severe antibiotic resistance profile of this mutant (Figure 7d and Table S3).

### 3.9. Trajectory Analysis of the AMP Insertion Pathway and Bilayer Interface Partitioning

**Figure 8:**
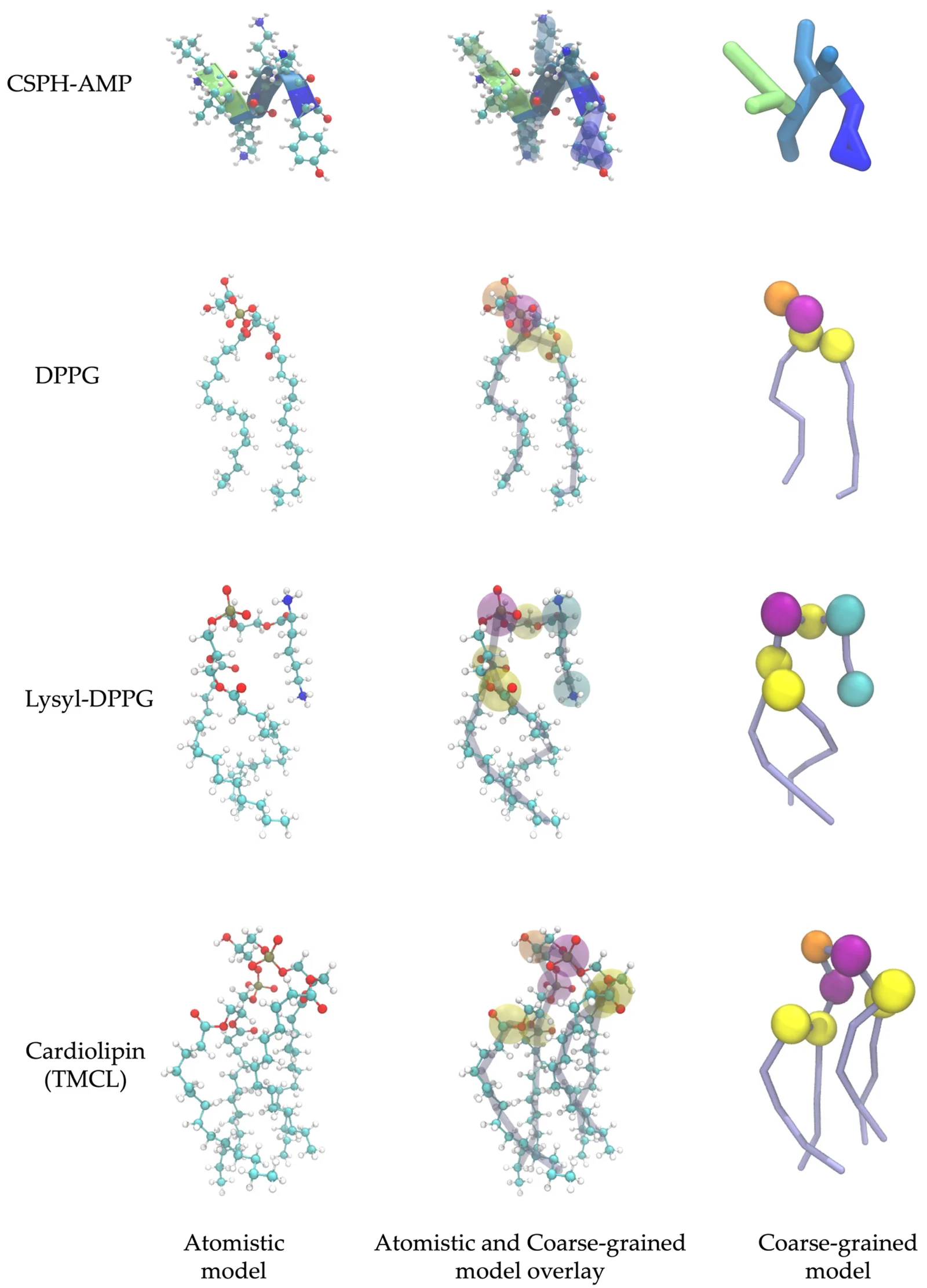
Coarse-grained representation of CSPH-AMP and the simulated membrane system. The atomistic models of CSPH-AMP and the simulated membrane, initially represented using a CPK coloring style, were converted into coarse-grained beads parameterized according to the Martini force field. In the peptide color scheme, green represents hydrophobic leucine residues, sky blue denotes positively charged lysine residues, and blue indicates the hydrophobic tyrosine residue. For the phospholipid model, the purple head groups represent negatively charged phosphate moieties, the orange beads represent the polar hydroxyl groups, the yellow beads denote the polar glycerol regions, and the ocean blue beads indicate positively charged lysyl moieties. Finally, the periwinkle blue tail sections represent the hydrophobic lipid tails.

To characterize the molecular mechanism of AMP interactions, coarse-grained MD simulations were performed using a mimetic *S. aureus* membrane bilayer composed of 1,2-O-dipalmitoyl-sn-glycero-3-phospho-(1′-rac-glycerol) (DPPG) at 40%, 1,2-O-dipalmitoyl-sn-glycero-3-phospho-rac-(3-lysyl(1′-glycerol)) (Lysyl-DPPG) at 52%, and 1,1′,2,2′-tetramyristoyl cardiolipin (TMCL) at 8% [45,46]. The atomistic models of CSPH-AMP and the simulated membrane phospholipid molecules were converted into coarse-grained beads and parameterized according to the Martini force field (Figure 8). The initial right-handed α-helical conformation of the CSPH-AMP model was constructed using Discovery Studio (version 2025). This constructed AMP was first solvated and simulated in a hydration box to achieve a biologically relevant aqueous conformation prior to its introduction to the membrane system. The simulated bacterial membrane was constructed with a specific lipid composition that accurately reflects the complex anionic environment and net charge density characteristic of the *S. aureus* cell membrane.

**Figure 9:**
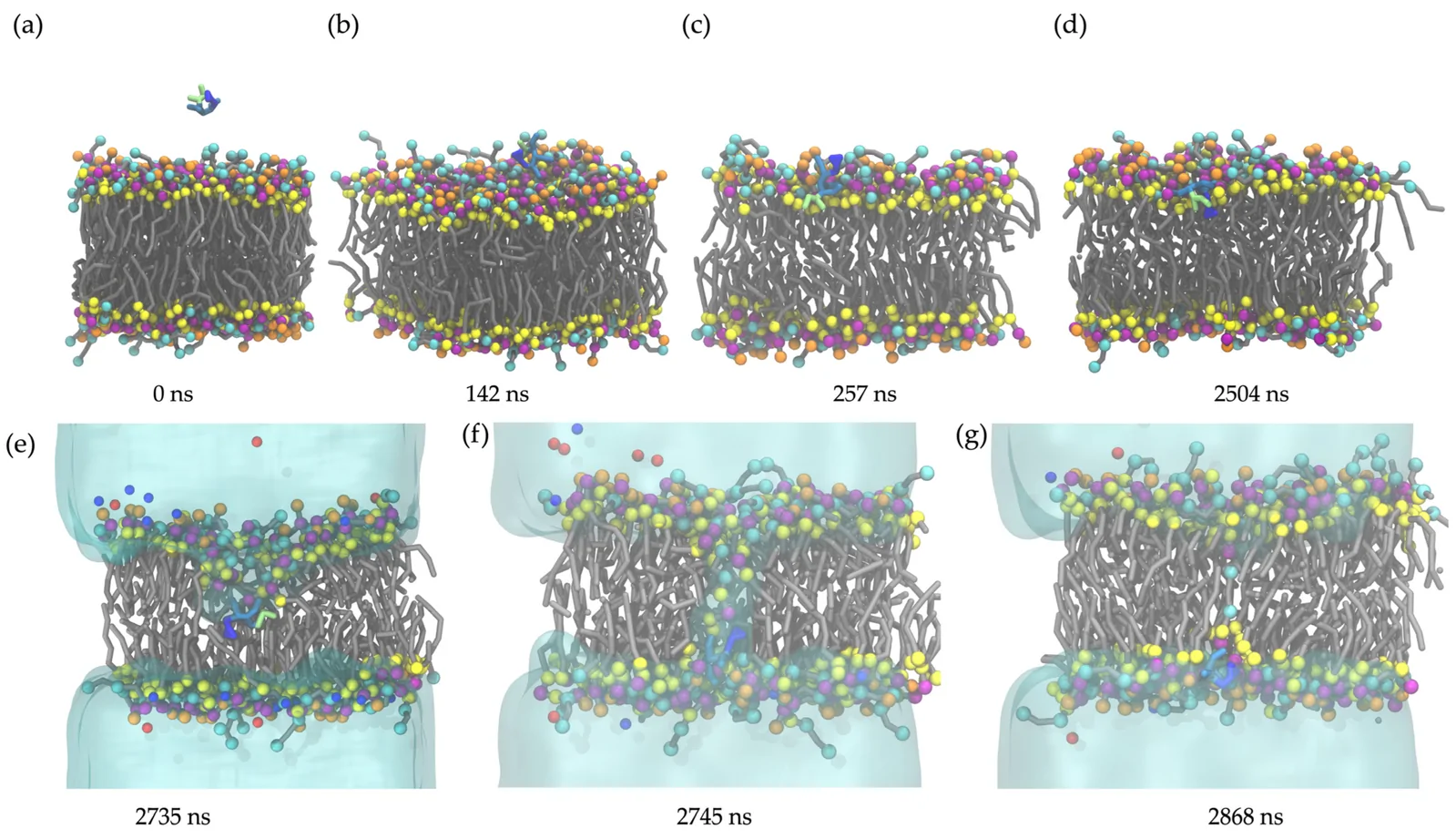
Structural evolution of the peptide–lipid system over a 2868-ns trajectory. Sequential snapshots (**a**–**d**) demonstrate the spontaneous binding of CSPH-AMP to the simulated *S. aureus* membrane surface from 0 to 2504 ns, at which point it reaches a steady state. Sampling of the reaction coordinate along the *z*-axis was performed using the adaptive biasing force (ABF) method. The snapshots (**e**–**g**) captured during biased pulling display the peptide’s orientation during membrane penetration, as well as the membrane distortion that facilitates water leakage into the formed pore. The color representations for the peptide and lipids are identical to those detailed in Figure 8.

Analysis of the simulation trajectories over a total period of 2868 ns revealed a clear conformational evolution of CSPH-AMP, starting from its initial state at 0 ns (Figure 9a). By 142 ns, the peptide had successfully associated with the membrane surface, an interaction mediated primarily by the lysine residue beads (Figure 9b). The peptide then underwent a spontaneous structural transition at 257 ns (Figure 9c), progressing from this surface-bound state to a stable, deeply embedded configuration by 2504 ns (Figure 9d). Following the application of an adaptive bias to pull the peptide along the *z*-axis to sample the reaction coordinate, the simulation captured its complete penetration through the hydrophobic core at 2735 ns (Figure 9e). During this translocation, the positively charged lysine beads electrostatically dragged the negatively charged phospholipids into the core at 2745 ns (Figure 9f). Ultimately, the lysine residues successfully reached the distal phospholipid headgroup region at 2868 ns (Figure 9g). This full-depth translocation suggested a robust interaction between the peptide and the bilayer. Such successful transmembrane insertion and the subsequent dynamic rearrangement of the surrounding lipid headgroups provided strong computational evidence for a membrane-disrupting mechanism, likely involving pore formation and the induction of water leakage across the membrane bilayer. Furthermore, the dynamic process of the peptide anchoring and inserting into the lipid bilayer over the course of the simulation can be visualized in Supplementary Video S1.

### 3.10. Mean Square Displacement (MSD) and Root Mean Square Fluctuation (RMSF) Profiles of the Membrane-Bound Peptide

**Figure 10:**
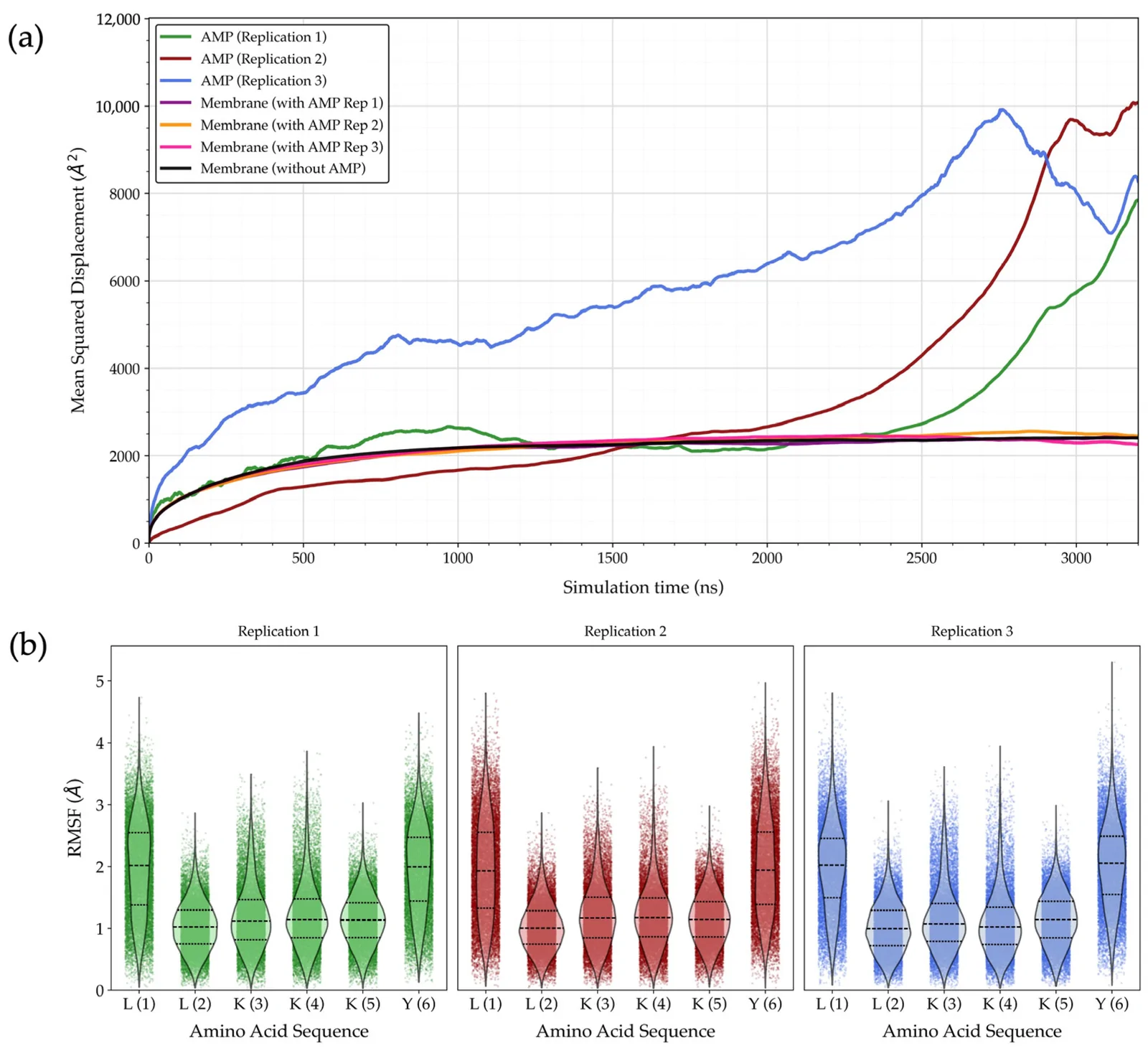
Dynamic properties and residue-specific flexibility of the peptide–lipid system. Mean square displacement (MSD) of CSPH-AMP and lipid bilayer as a function of simulation time (**a**). Root mean square fluctuation (RMSF) profile of CSPH-AMP, providing a detailed map of residue-specific fluctuations and local conformational flexibility within the membrane interface (**b**). In the violin plots, the horizontal lines within each distribution indicate the median and interquartile ranges.

The mean square displacement (MSD) was calculated as a function of simulation time to quantify the spatial displacement of each component of interest. The MSD values for the phospholipid bilayer remained remarkably stable throughout the initial trajectory, confirming the structural integrity of the bacterial membrane prior to peptide insertion. In contrast, the MSD of the peptide showed a steady increase during the first 1000 ns as it sampled various conformations, eventually reaching a plateau between 1000 ns and 2500 ns. This plateau indicated that the system had reached a steady state, with the peptide maintaining a stable, and reached the equilibrated state adsorbed to the membrane surface. After 2500 ns, the simulation system was required to pull the peptide molecule into membrane by adaptive biasing force (ABF) algorithm to collect the information of Gibbs free energy by collective variables for potential of mean force (PMF) (Figure 10a) [47].

The RMSF profile provided a detailed map of the residue-specific interactions between the peptide and the bacterial membrane. The central residues, including L2, K3, K4, and K5, exhibited significantly lower RMSF values, indicating a loss of conformational mobility (Figure 10b). This rigidity suggested that these specific amino acids served as the primary anchoring domain, where the hydrophobic leucine and tyrosine residues were deeply stabilized by the lipid hydrophobic core, while the lysine residues favorably interacted with the phosphate headgroups of the phospholipids. In contrast, the terminal residues L1 and Y6 showed higher RMSF values, characteristic of terminal mobility. This heightened flexibility indicated that the peptide termini were not conformationally restricted, leaving them free to fluctuate during membrane binding. This gradient of flexibility, transitioning from a rigid central core to mobile termini, directly correlated with the highly bent state of the peptide. Such a conformation perfectly aligned with the structural requirements of the toroidal pore mechanism.

### 3.11. Mechanistic Insights into Peptide Translocation: Thermodynamics, Orientation, and Structural Transitions

**Figure 11:**
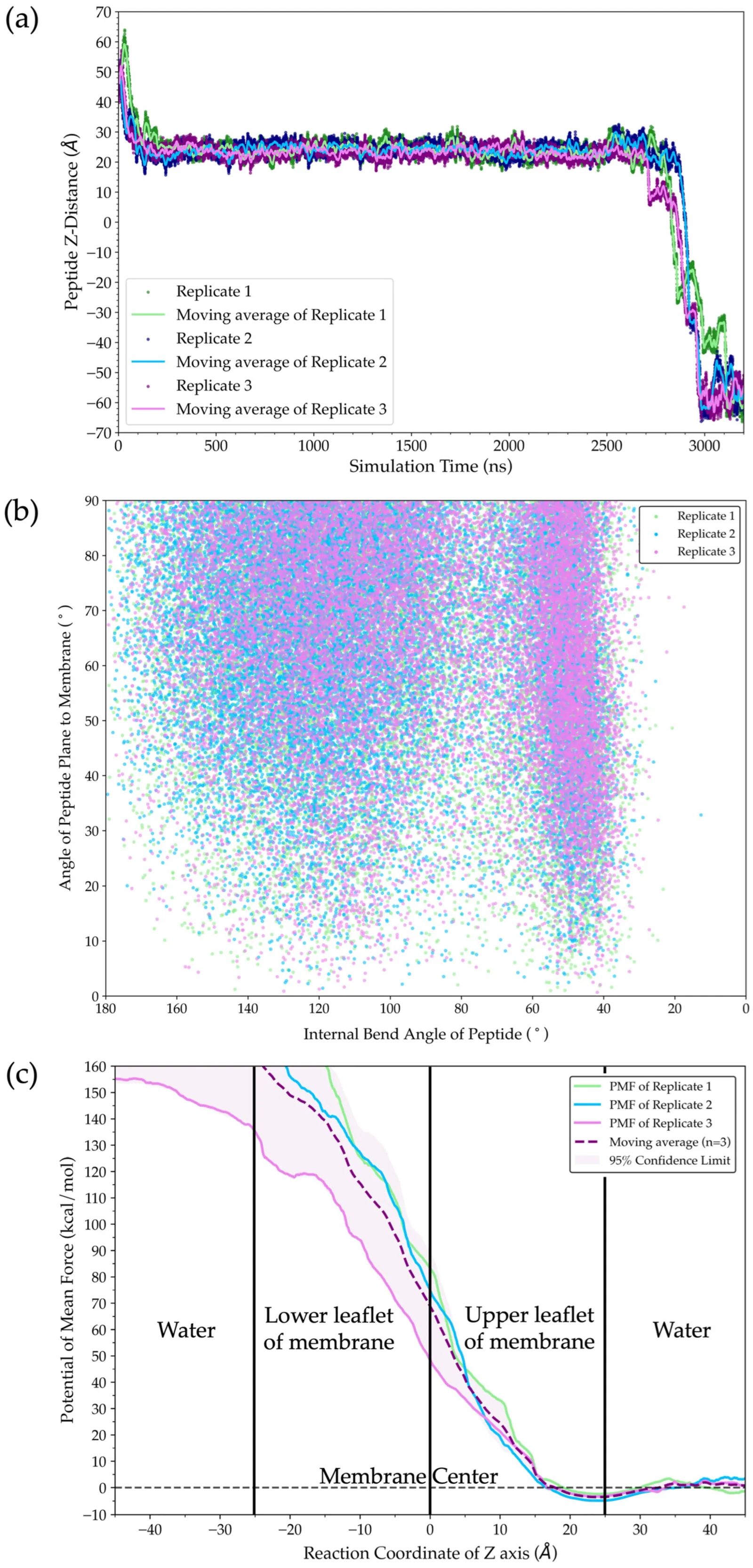
Kinetic, structural, and thermodynamic characterization of CSPH-AMP membrane insertion. Time-dependent *z*-distance between the peptide center of mass and the membrane bilayer midplane, illustrating the transition from surface adsorption to transmembrane insertion (**a**). Correlation between the internal peptide bend angle and the angle of the peptide plane relative to the membrane normal (**b**). Potential of mean force (PMF) profile along the reaction coordinate of the *z*-axis, representing the free energy landscape of the peptide translocation process (**c**).

Analysis of the *z*-distance further clarified the peptide–membrane interaction kinetics. At 50 ns, a high *z*-distance of approximately 50 Å above the membrane confirmed that the peptide remained in the aqueous phase, diffusing freely and ensuring that the initial peptide–lipid contacts were captured without starting bias. A rapid decrease in the *z*-distance occurred at approximately 100 ns, followed by a stabilization at approximately 25 Å. This suggests that the peptide was securely adsorbed onto the phosphate headgroups, primarily driven by electrostatic interactions (Figure 11a). Following the equilibration phase (at 2500 ns), the ABF method was initiated to drive the peptide across the membrane. This resulted in a sharp increase in the peptide’s MSD and a corresponding drop in *z*-distance, marking the transition from surface adsorption to deep embedding within the hydrophobic core of the lipid bilayer (Figure 11a).

The relationship between the internal bend angle of the peptide and the angle of the peptide plane relative to the membrane normal was analyzed to determine the structural requirements for membrane penetration. The data indicated that successful insertion occurred within two specific conformational states, namely a highly bent state with an internal angle of 40–60° paired with a tilt angle of 10–90°, and a moderately bent state of 100–140° with a tilt range of 30–90°. The peptide translocated by adopting a specifically tilted and bent configuration to bypass the lipid headgroup barrier. This coordinated shift in both geometry and spatial orientation provided strong structural evidence for a toroidal pore mechanism, as the bending allowed the peptide to stabilize the high-curvature lipid-peptide interface required for a functional transmembrane pore (Figure 11b).

The free energy of the peptide–membrane interaction was characterized by plotting the PMF against the reaction coordinate or the *z*-distance from the center of the bacterial membrane (Figure 11c). At a distance of 30–40 Å, the PMF remained at zero, representing the reference state in bulk water. In this region, the peptide experienced no significant electrostatic interactions with the bilayer. As the peptide approached the membrane interface (*z* = 25 Å), the PMF reached the minimum (negative) values, indicating that adsorption to the phospholipid headgroups was a thermodynamically spontaneous process. This initial binding occurred at approximately 100 ns of the trajectory.

To investigate the translocation process, ABF was applied. At approximately 2500 ns, the PMF reached 70 kcal/mol at the bilayer center (*z* = 0 Å). This correlates with the structural disruption observed at 2735 ns (Figure 9e), where the peptide achieved full insertion into the bilayer midplane, leading to the loss of membrane integrity. During this transition, the peptide pulled several lipid headgroups into the hydrophobic interior, creating a water-filled channel. This led to membrane leakage, confirming that the antimicrobial mechanism proceeded via the toroidal pore model. As the peptide translocated further into the lower leaflet, the PMF continued to increase, requiring a free-energy input of of 135 kcal/mol. This high energy requirement suggested that while the initial membrane penetration occurred, the complete translocation into the intracellular aqueous phase at nearly 3000 ns (Figure 9g) represented the most energetically demanding stage of the permeabilization pathway (Figure 11c).

### 3.12. Spatiotemporal Characterization of Peptide–Membrane Interactions and Induced Bilayer Deformation

**Figure 12:**
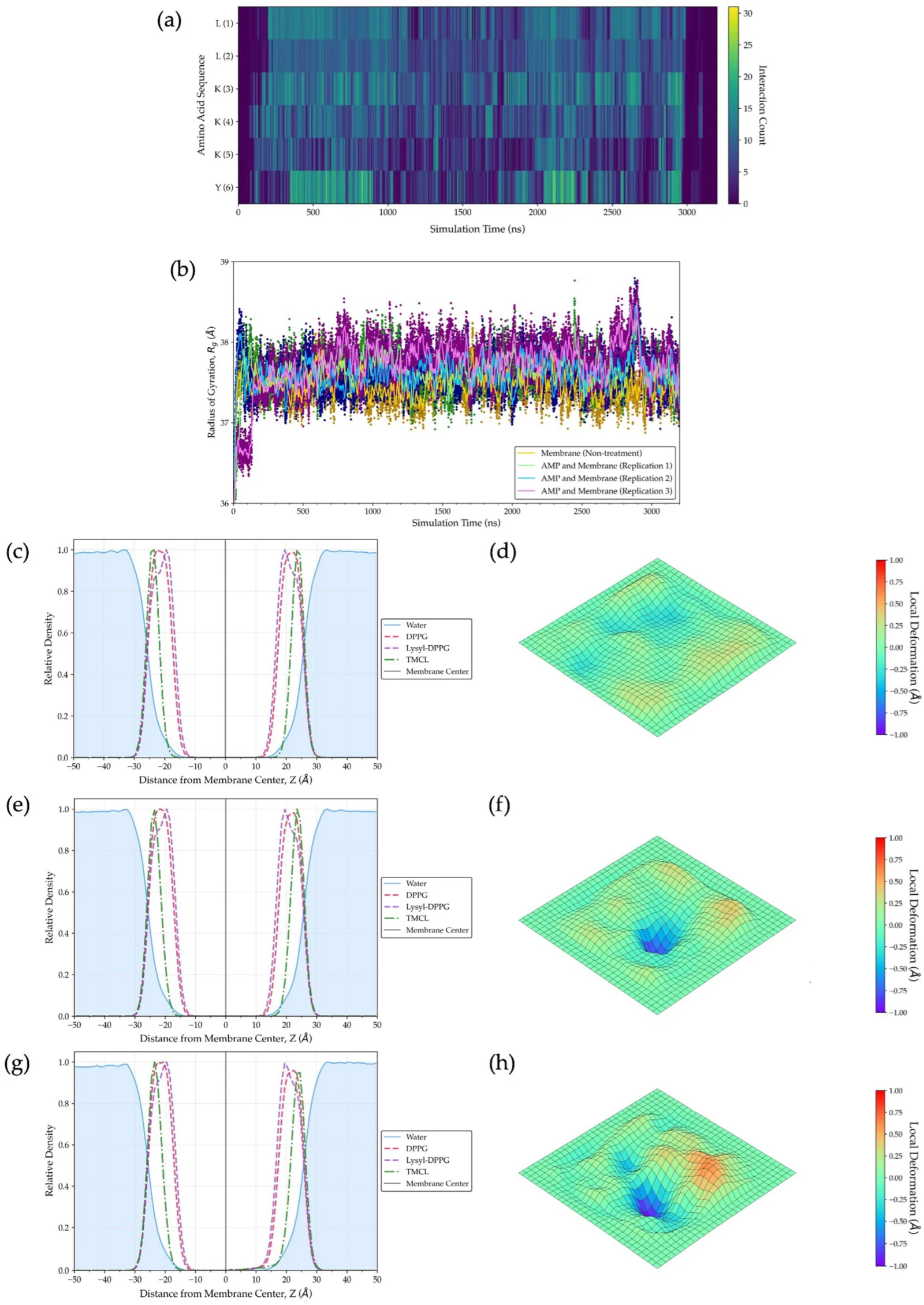
The contact heat map of peptide–membrane interactions. The plot illustrates the interaction frequency (interaction count) for each amino acid residue with the bacterial membrane as a function of simulation time (**a**). Comparative radius of gyration (*R_g_*) analysis of peptide–membrane dynamics. The stable *R_g_* values for the plain membrane serve as a structural control for the duration of the simulation, with the scattered dots representing the raw data points plotted alongside their corresponding moving average lines (window size = 50) (**b**). Evolution of membrane mass density profiles during peptide translocation. The relationship between the distance from the membrane center (*z* = 0) and the relative density of the membrane components is shown for the control membrane (**c**), initial adsorption at 1392 ns (**e**), and post-ABF translocation at 2868 ns (**g**). The shifting peaks indicate the structural perturbation of lipid headgroups and water molecules as the peptide penetrates the hydrophobic core. Evolution of membrane surface topography and local deformation induced by CSPH-AMP. Three-dimensional surface maps illustrate the bacterial membrane at the initial control state (**d**), during peptide adsorption at 1392 ns (**f**), and following ABF-driven translocation at 2868 ns (**h**). The color scale represents deformation by vertical displacement relative to the bilayer midplane (*z* = 0).

The contact heat map (Figure 12a) illustrates the spatiotemporal interaction frequency between individual amino acid residues and the bacterial membrane. This analysis identified the primary anchoring residues and the sequence of interaction throughout the simulation trajectory. At approximately 100 ns, Y6 established the initial contact, maintaining the highest interaction count until 800 ns. Within the 1000–2000 ns interval, the binding was dominated by K3, K4, and Y6, representing the main stabilizing residues. Between 2000 and 2500 ns, Y6 remained the most frequent interactor. Following the application of ABF at 2500 ns, K3 and Y6 emerged as the primary residues facilitating the translocation and pore-formation process. This study highlighted the critical roles of the positively charged lysine residues and the hydrophobic aromatic tyrosine residue in the membrane-binding process.

The structural evolution of the peptide–membrane system was evaluated by monitoring the radius of gyration (*R_g_*) and the tilt orientation of the lipid bilayers over the simulation trajectory (Figure 12b). The *R_g_* values for the phospholipid membrane without the peptide remained significantly lower than those of the peptide–membrane system and exhibited only minor fluctuations throughout the simulations. This stability serves as an internal control, confirming that the membrane maintained its structural integrity prior to the interaction with the peptide. Compared to the control membrane, the lipid tilt angles showed a significant increase, indicating that CSPH-AMP induced substantial localized disorder within the bacterial membrane interface.

Upon the application of the ABF at 2500 ns, a notable increase in the peptide’s *R_g_* was observed. This expansion correlates with the conformational transition from a compact surface-bound state to a more extended configuration required to span the bilayer. Simultaneously, the orientation of the lipid molecules relative to the membrane normal (*z*-axis) was analyzed to quantify membrane disturbance. This perturbation was further amplified during the ABF-driven translocation.

The structural integrity of the multicomponent bacterial membrane comprising DPPG, Lysyl-DPPG, and TMCL was evaluated by analyzing the normalized relative densities across the simulation trajectory (Figure 12c,e,g). At the initial state (t = 0 ns), the lipids exhibited a well-ordered bilayer distribution with a relative density of 1.0 at the headgroup regions. Upon peptide interaction at 1392 ns, a localized reduction in the relative density of the DPPG headgroups was observed, signaling the onset of lipid displacement as the AMP began to penetrate the interface. By 2868 ns, this structural disruption was further amplified, with a significant decrease in the density of both DPPG and TMCL components within the headgroup regions. Concurrently, the density profiles for water molecules (indicated by the blue line) showed a marked shift toward the membrane center at both 1392 ns and 2868 ns. This inward migration of water provided direct computational evidence that the AMP disrupted the hydrophobic core of the bilayer (Figure 12c,e,g).

The structural impact of CSPH-AMP on the bacterial membrane was quantified by analyzing the local deformation at key simulation timestamps (1392 ns and 2868 ns), using the undisturbed membrane without the peptide as a reference. At 1392 ns, as the peptide adsorbed and began to penetrate the upper phospholipid headgroups, the membrane exhibited significant local deformation. Specifically, regions of negative deviation (values < 0.00) indicated membrane indentation toward the bilayer midplane as the peptide pushed inward. Conversely, adjacent regions showed a positive deviation (values > 0.00), representing an elevation of lipid headgroups as they were displaced by the peptide’s bulk. By 2868 ns, when the peptide achieved full insertion into the lower phospholipid headgroups, these topographic perturbations became significantly more pronounced. The increased local deformation indicated that the peptide actively deformed the lipids from both leaflets, leading to a permanent loss of membrane integrity (Figure 12d,f,h).

## 4. Discussion

The urgent threat of antimicrobial resistance, particularly from MRSA, necessitates the discovery of novel therapeutics to mitigate a projected millions of attributable deaths by 2050 [1]. To address this critical gap, the scientific community is actively exploring diverse, non-traditional antimicrobial modalities. Recent advancements have demonstrated significant potential in alternative strategies such as bacteriophage therapy, metal nanoparticles, and cold atmospheric plasma treatments [48,49,50,51]. While these approaches offer innovative mechanisms to overcome resistance, such as the high target specificity of phages or the broad-spectrum physical disruption induced by plasma and metal nanoparticles, their clinical translation can be hindered by practical challenges, including narrow host ranges, rapid immune clearance, formulation instability, or inherent mammalian cytotoxicity. As a viable alternative, the present study capitalized on the vast, untapped biological potential of industrial rubber manufacturing waste. The innate immune arsenal of *H. brasiliensis* latex is well-documented, primarily driven by natively expressed defense proteins. The most prominent is Hevein, a 4.7 kDa cysteine-rich peptide that exerts potent antifungal activity by binding to structural chitin, while also inhibiting opportunistic human oral pathogens (e.g., *Candida* species). Another major native constituent is Hev b5, a 16 kDa highly acidic latex protein. Unlike typical cationic AMPs, Hev b5 is anionic and relies on divalent metal cations (such as Ca^2+^ or Zn^2+^) to form electrostatic salt bridges that disrupt bacterial membranes. The latex is further fortified by synergistic enzymes, including chitinase-active hevamines and β-1,3-glucanases, as well as protease inhibitors (HPI) that protect these native defenses from degradation [2,3,4]. However, these natively expressed macromolecules differ fundamentally in structure, size, and mechanism from CSPH-AMP, a short, highly cationic (+3) cryptic hexapeptide that does not exist freely in the latex but is exclusively liberated through the targeted enzymatic hydrolysis of C-serum waste.

This study demonstrated that the latex wastewater contains AMPs following protease-mediated hydrolysis. The obtained AMP exhibited membrane-disruptive activity. Enzymatic hydrolysis is the most prevalent strategy for producing bioactive peptides due to its high reproducibility, scalability, and precise process control achieved by selecting specific proteases. This targeted cleavage yields peptides with diverse functional properties, including antimicrobial, antihypertensive, and antioxidant activities [9,52]. In the current industrial landscape, there is a growing emphasis on the environmental and socio-economic impacts of protein sourcing. This has shifted research focus toward sustainable alternatives, such as plant-based proteins and industrial byproducts, which offer significant ecological advantages over traditional animal-derived sources [53]. Natural proteins represent a critical reservoir of bioactive peptides, which remain entirely latent and inactive within the primary sequence of their parent proteins. These cryptic bioactivities are only expressed once the polypeptide chains are cleaved, a process typically driven by gastrointestinal digestion, microbial fermentation, or targeted enzymatic hydrolysis [54]. A major advantage of generating AMPs through enzymatic hydrolysis is the ability to significantly potentiate the inherent antimicrobial activity of a protein beyond its native limitations [55]. Beyond enhancing bioactivity, the enzymatic hydrolysis of proteins supports sustainable industrial upcycling by transforming abundant, low-cost agricultural and fisheries byproducts into high-value functional ingredients [56].

This research provides a roadmap for the sustainable discovery of new antibiotics. The antimicrobial activity and inhibitory spectrum of the proteinase K-derived hydrolysates can be primarily attributed to the enzyme’s distinct cleavage profile, which is particularly effective against the complex proteinaceous matrix of *H. brasiliensis* latex wastewater. Acting as a non-specific serine endopeptidase, proteinase K catalyzes the breakdown of proteins by targeting the carboxylic side of hydrophobic, aliphatic, and aromatic amino acid residues [12]. The identification of the kinesin-like protein KIN-14S (XP_021653015.2) as the parent protein of CSPH-AMP validates *H. brasiliensis* C-serum as a robust source of cryptic AMPs [43]. The release of the LLKKKY sequence is supported by the well-characterized cleavage profile of proteinase K. Within the parent protein structure, this hexapeptide is positioned between aromatic residues (phenylalanine and tyrosine). Because proteinase K preferentially hydrolyzes peptide bonds adjacent to these aromatic sites, the enzyme was highly effective at liberating this cryptic bioactive sequence from the larger, inactive precursor protein [57]. The generation of the active CSPH-AMP (LLKKKY) from a kinesin-like parent protein underscores the critical role of targeted enzymatic hydrolysis in unlocking encrypted bioactive peptides (cryptides) from discarded natural rubber latex proteins [58]. The modifications responsible for the observed antimicrobial effects begin during the initial protein preparation procedure. Prior to protease digestion, the complex proteinaceous matrix of *H. brasiliensis* C-serum wastewater is subjected to heat-induced precipitation (95 °C). This thermal treatment effectively denatures the complex folded tertiary structures of the proteins. Consequently, the isolated protein precipitate consists primarily of denatured, unfolded polypeptide chains, which eliminates steric hindrance and renders the primary structure highly prone to rapid enzymatic digestion [59]. The antimicrobial activity of the resulting hydrolysates can be primarily attributed to the distinct cleavage profile of Proteinase K. Acting as a non-specific serine endopeptidase, Proteinase K catalyzes the breakdown of proteins by targeting the carboxylic side of hydrophobic, aliphatic, and aromatic amino acid residues [12,60]. The identification of the kinesin-like protein KIN-14S (XP_021653015.2) as the parent protein of CSPH-AMP perfectly validates *H. brasiliensis* C-serum as a robust source of cryptic AMPs. Within this parent protein structure, the hexapeptide sequence is positioned directly between aromatic residues (phenylalanine and tyrosine). Because Proteinase K preferentially hydrolyzes peptide bonds adjacent to these specific aromatic sites, the enzyme is highly effective at liberating this highly cationic, membrane-disruptive sequence from the larger, inactive precursor protein. Furthermore, it is critical to note that protease digestion is a highly dynamic process; active peptide sequences are continuously released while other precursor proteins are simultaneously degraded [61]. Although the liberated LLKKKY sequence itself contains a C-terminal aromatic residue (tyrosine) making it theoretically susceptible to further degradation by Proteinase K into smaller, inactive fragments [12,60]. The peptide retained its antimicrobial activity following the digestion process. The successful identification of this sustained bioactivity was achieved through a time-dependent, bioassay-guided discovery approach. By systematically tracking the digestion time and evaluating the antimicrobial activity of the hydrolysates at specific intervals, we were able to capture the functional LLKKKY sequence at its optimal kinetic release point, prior to complete enzymatic degradation [58]. Ultimately, this detailed biochemical characterization provides a robust roadmap for the sustainable discovery of new membrane-active antibiotics from agricultural waste streams [62]. The antibacterial potential of CSPH-AMP is supported by its sequence homology with established AMPs in the APD6 database. Specifically, CSPH-AMP shares 62.5% sequence similarity with the de novo designed peptides L4K3W^4^ (LLKWLKKL) and L3K3W^4^ (LLKWLKK). These homologous peptides exhibit comparable antibacterial efficacy against *S. aureus*, with reported MICs of 25 µg/mL and 200 µg/mL, respectively. Furthermore, a physicochemical comparison reveals similarities between these reference peptides and CSPH-AMP. Although the homologues are slightly longer (8 and 7 amino acids, respectively) than the 6-residue CSPH-AMP, all three peptides share an identical net charge of +3. Their grand average of hydropathicity (GRAVY) scores are also within a comparable range: L4K3W^4^ and L3K3W^4^ score 0.325 and −0.171, respectively, while CSPH-AMP is slightly more hydrophilic with a score of −0.9. To further predict peptide–membrane interactions, Wimley–White whole-residue hydrophobicity was assessed by calculating the Gibbs free energy of binding to a neutral phosphatidylcholine (PC) lipid bilayer. The free energy values for L4K3W^4^ (−1.12 kcal/mol) and L3K3W^4^ (−0.56 kcal/mol) provide a thermodynamic baseline for comparison with CSPH-AMP (0.91 kcal/mol) [63]. Importantly, this positive difference in calculated binding energy indicates that CSPH-AMP preferentially targets the more hydrophilic, negatively charged phospholipid bilayers characteristic of bacterial membranes (e.g., those containing dipalmitoylphosphatidylglycerol; PG) rather than the neutral phospholipids typical of mammalian cell membranes (e.g., those containing phosphatidylcholine; PC) [64,65,66]. The thermodynamic and structural profile of CSPH-AMP closely mirrors the established heptapeptide KKKLAYA, which shares an identical +3 net charge, a comparable GRAVY score (−0.8), and a positive Wimley–White value (1.81 kcal/mol) alongside potent inhibitory activity against *Staphylococcus* species [67]. Including, surface attraction, peptide efficacy depends on amphipathic partitioning into the lipid bilayer. The GRAVY score of CSPH-AMP (−0.90) indicates sufficient hydrophilicity for aqueous solubility, positioning it as a functional intermediate between the homologous L3K3W^4^ (−0.171) and the highly potent synthetic peptide P9-4 (−1.31) [68]. Ultimately, this structural and physicochemical alignment with established APD6, DRAMP, and DBAASP peptides supports that the LLKKKY sequence exhibits antibacterial activity against *S. aureus* and MRSA.

The broad cleavage profile of proteinase K is highly advantageous for the generation of AMPs from industrial effluents, as it facilitates the breakdown of diverse residual proteins found in rubber latex, thereby liberating cryptic bioactive sequences. Developing scalable batch processes for protein hydrolysates is a crucial strategy for upcycling and adding value to byproducts in the natural product industry. Furthermore, the valorization of protein waste into AMPs can generate new revenue streams for manufacturers. For instance, previous reports have demonstrated that animal-derived byproducts, such as anchovy cooking wastewater containing 5.07 g/L of protein, can be converted by endopeptidases (e.g., Alcalase/Protamex) into protein hydrolysates exhibiting antibacterial activity at 12.10 μg/mL [69]. Similarly, the present study is grounded in the bio-circular economy framework, successfully upcycling the residual proteins in C-serum wastewater from rubber latex production. This study paves the way for sustainable resource recovery by establishing a feasible approach to upcycling plant protein waste from natural rubber manufacturing.

The MIC and MBC values of CSPH-AMP proposed insights into its use as an alternative antibacterial agent. An MBC/MIC ratio of 4 in µg/mL range was a hallmark of bactericidal agents, distinguishing the AMP from bacteriostatic compounds that merely inhibit growth without achieving cell death. This profile suggests that the AMP operates via a direct killing mechanism—likely membrane disruption—which successfully bypasses established resistance pathways [70]. Highly evolved innate immune peptides, such as mammalian LL-37, amphibian temporins, and clinical synthetic analogs like pexiganan, frequently establish the benchmark for potency with MICs ranging from 4 to 64 µg/mL [71,72,73]. Similarly, native plant defense peptides such as cyclotides, 2S albumins, lipid transfer proteins, hevein-like proteins, knotins, snakins, and purothionins typically exhibit antimicrobial activity in the micromolar range [74,75]. Beyond planktonic inhibition, CSPH-AMP demonstrated significant antibiofilm efficacy against MRSA, the effective concentrations reflecting increase over its MIC. This proportional shift is highly consistent with the pharmacodynamic profiles of established clinical benchmarks, such as amphibian temporins and the synthetic analog pexiganan, which similarly require exponentially higher doses to overcome electrostatic sequestration within the dense extracellular polymeric substance (EPS) matrix [76,77]. In contrast, human LL-37 inhibits biofilm formation at concentrations lower than those required to kill planktonic cells [74]. Furthermore, CSPH-AMP exhibits distinct biophysical properties compared to natively expressed plant defense macromolecules, including plant defensins, cyclotides, 2S albumins, lipid transfer proteins, hevein-like proteins, knotins, snakins, and purothionins [74,75].

CSPH-AMP induced a concentration-dependent increase in fluorescence in the SYTOX Green assay, indicating significant membrane compromise and the formation of SYTOX Green-DNA complexes. While both strains showed comparable permeabilization at lower concentrations, MRSA exhibited a significantly more pronounced response at 8× MIC, suggesting a heightened susceptibility to membrane-mediated disruption at elevated concentrations. Ultrastructural analysis via SEM revealed that both *S. aureus* TISTR 517 and MRSA strain 2468 exhibited localized indentations comparable to the positive control vancomycin. The morphological distortion confirms that CSPH-AMP operates via a membrane-active mechanism.

The antibacterial potential of CSPH-AMP is strongly supported by its high resilience to diverse environmental stresses. The peptide’s thermal stability at temperatures up to 100 °C is particularly noteworthy. While the loss of activity under autoclave conditions (121 °C and high pressure) defined a threshold for its structural integrity, the peptide’s ability to withstand boiling makes it highly suitable for diverse industrial applications. CSPH-AMP remained stable against proteolytic enzymes for the first 1 h. It exhibited a gradual reduction in potency over a 12 h incubation, which is a common characteristic of linear peptides. However, this initial stability window is sufficient to achieve effective localized concentrations prior to clearance. This resilience aligns with previous reports indicating that highly cationic AMPs rich in lysine and leucine residues exhibit robust thermotolerance and protease resistance [78]. The degradation kinetics across these three distinct proteases can be directly attributed to the specific amino acid composition of the LLKKKY hexapeptide. Because CSPH-AMP is an ultrashort, linear sequence devoid of a protective secondary or tertiary structure, its primary peptide bonds are highly exposed to enzymatic attack [79]. Crucially, the hexapeptide sequence provides an ideal, highly concentrated substrate motif for all three tested enzymes. The core poly-lysine motif renders the peptide highly susceptible to trypsin, which strictly cleaves at the carboxyl side of basic amino acids [80]. Concurrently, the terminal leucine and tyrosine residues serve as primary cleavage targets for both α-chymotrypsin (which favors large hydrophobic and aromatic residues) [81] and proteinase K (a broad-spectrum protease with a preference for aliphatic and aromatic groups) [12]. Consequently, regardless of the specific protease class applied, the enzyme is presented with immediate, unimpeded access to its preferred cleavage sites. The short chain length of the hexapeptide may unrecognized by enzymatic active site, leading to a slower catalytic turnover rate compared to the rapid degradation typically observed in larger, denatured proteins with flexible loops [82]. CSPH-AMP exhibited synergistic antibacterial activity against MRSA when combined with SDS or Triton X-100. This aligns with previous studies on the cationic peptide G3 (G(IIKK)_3_I-NH_2_), which demonstrated that anionic and non-ionic surfactants enhance antimicrobial activity by forming peptide–surfactant complexes. Conversely, the introduction of a cationic surfactant (CTAB) completely abolished the peptide’s antimicrobial activity [83]. Furthermore, CSPH-AMP demonstrated enhanced antibacterial activity when incubated under acidic conditions. This property suggests that CSPH-AMP could be particularly effective in acidic physiological environments, such as the gastric mucosa or infected wound sites. In these environments, bacterial pathogens frequently secrete metabolites, including acetic, lactic, and malic acids, thereby establishing a localized acidic microenvironment [84]. The enhanced antimicrobial activity of the peptide in a low-pH environment is likely driven by an increase in its net positive charge, which strengthens its electrostatic binding to the anionic bacterial membrane [85].

Proteomic analysis of *S. aureus* and MRSA following CSPH-AMP treatment provides crucial evidence to investigate the proposed mechanisms underlying the bacterial response to this AMP. Previous proteomic studies have demonstrated that AMP-induced membrane damage triggers a robust stress response, shifting cellular machinery into a survival mode. For example, following treatment with AMPs (such as the SK1260 and R4F4 peptides), the upregulation of molybdopterin cofactor biosynthesis protein A (*moaA*), energy breakdown enzymes (*fda*), and pH maintenance proteins (*argF-2*) indicates a cellular stress response. This response shifts the cellular system toward virulence while compensating for energy depletion and maintaining the pH of cellular glycolysis during membrane damage [86,87,88]. Direct damage to the bacterial membrane—whether caused by potassium carrier ionophores like valinomycin or cationic AMPs—induces the selective accumulation of superoxide. Consequently, these membrane-disrupting agents specifically trigger the upregulation of superoxide dismutase (*SodA*), bypassing other reactive oxygen species (ROS) detoxification pathways [89]. Under AMP attack, bacteria coordinate a multi-tiered defense integrating ion homeostasis, structural repair, and metabolic priming to survive envelope stress. To restore collapsed membrane potentials and stabilize internal ionic environments, cells upregulate transmembrane transporters like the FecCD permease (*SAR2266*), ABC-2 type systems (*SAR2595*), and the cobalt transporter (*ecfT*). This strategy directly mirrors the induction of the Na^+^/H^+^ antiporter subunit MnhC2 under SK1260 stress in *S. aureus* [88], the *corA*/*pit* ion transport network in *Escherichia coli* [87], and the *fhuA* iron-siderophore transporter in *Chromobacterium violaceum* [90]. Simultaneously, structural and genomic integrity is preserved by upregulating the penicillin-binding protein PBP2B (*pbpF*) for peptidoglycan repair and DNA ligase (*ligA*) to mend DNA strand breaks caused by rapid, AMP-induced superoxide accumulation. This echoes the cell wall stress responses regulated by major ECF sigma factors (such as SigM and SigX) in *Bacillus subtilis* [91] and envelope modifications in *Listeria monocytogenes* [92]. Finally, metabolic networks are rewired via *ugpQ* to provide lipid precursors for protective bilayer remodeling, and *pepA* to drive a peptide-salvage program. This generates free amino acids as alternative energy sources or osmotic buffers, strongly resembling the buffering arginine deiminase (ADI) pathway of *L. monocytogenes* [92] and the antibiotic-induced amino acid release (AIAAR) system of *B. subtilis* [91]. Consistent with the canonical action of most AMPs, the computational and proteomic data presented herein substantiate membrane disruption as the primary, initiating mode of action. However, the possibility of secondary intracellular targeting remains a highly relevant and promising avenue for future research. Subsequent structural investigations, including targeted molecular docking and in vitro protein interaction assays against the specific hub proteins identified in our PPI network, will be prioritized to determine whether CSPH-AMP exerts dual-mechanism inhibitory effects on the *S. aureus* intracellular pathways identified in this proteomic profile.

The integration of molecular dynamics (MD) simulations has become a pivotal approach in pharmaceutical research for elucidating the complex biophysical phenomena that govern peptide–membrane interactions [93]. While traditional assays can identify antimicrobial efficacy and membrane rupture, computational modeling provides high-resolution insights to capture the spatiotemporal evolution of the translocation process. By employing advanced sampling techniques such as adaptive biasing force (ABF), it is possible to map the thermodynamic landscape and determine the precise potential of mean force (PMF) required for a peptide to transition from surface adsorption to full-depth insertion. These simulations allow for the detailed analysis of residue-specific anchoring, as well as conformational plasticity including the radius of gyration and internal bending. Moreover, the resulting localized bilayer deformation offers a comprehensive atomic-level blueprint of the antimicrobial mechanism that complements the experimental observations. The MD simulation trajectories provide a high-resolution temporal map of the peptide’s interaction with the mimetic *S. aureus* membrane, transitioning from initial recruitment to complete transmembrane translocation. In this model, CSPH-AMP inserts into the membrane and associates with lipid molecules, causing the phospholipids to bend inward to form pore complexes. This proposed mechanism is driven by peptide interactions, which induce localized membrane curvature and allow both the AMPs and lipid headgroups to become deeply embedded within the hydrophobic core [26]. Specifically, the MD simulations provided the proposed membrane interaction model of toroidal pore formation where the AMP associated via its polar faces with the lipid headgroups, inducing a continuous bend of the monolayers from the membrane surface toward the interior. This structural transition causes the lipids to tilt from the lamellar normal, effectively connecting the proximal and distal leaflets. Consequently, the resulting aqueous pore is lined by a supramolecular assembly of both the inserted peptides and the intercalated lipid headgroups, creating a continuous hydrophilic bridge across the hydrophobic core of the bilayer [94,95]. This mechanism correlates with the observed structural evolution of the CSPH-AMP–lipid system captured in the MD simulation snapshots. These findings provide a molecular-level explanation for the high fluorescence observed in the SYTOX Green assays, as such transmembrane structures would create the necessary pathway for the influx of impermeable dyes. RMSF analysis confirmed that while the central domain serves as a stable anchor, the peptide termini remain relatively unconstrained, likely residing in the more fluid environment of the aqueous-lipid interface. This rigid-core and mobile-tail dynamic is biophysically significant, facilitating the highly bent conformation observed in the structural analysis. Such a conformation is a supporting feature of the proposed toroidal pore mechanism [96].

The physical impact on the membrane is evidenced by significant localized deformation and lipid displacement. The 3D topographic analysis reveals a pattern of indentation and localized lipid elevation. This is corroborated by the relative density profiles, which show a marked reduction in headgroup density (DPPG and TMCL) and a simultaneous inward migration of water toward the hydrophobic core. The pronounced reduction in lipid density within the headgroup region could be driven by the synergistic effects of physical peptide interaction and the concurrent influx of water molecules. As the peptide partitions into the membrane, water appears to penetrate the upper regions of the hydrocarbon acyl chains, leading to a marked increase in hydrogen bonding with the lipid components. This water penetration suggests strong electrostatic interactions between the peptide and the negatively charged phosphate groups of the DPPG molecules, potentially acting as the initial step in membrane destabilization [35]. Furthermore, the cationic CSPH-AMP likely interacts directly with TMCL (cardiolipin), a dimeric anionic phospholipid. This electrostatic attraction and subsequent peptide insertion could lead to the observed density reduction [97].

Predicting the membrane-insertion probability of diverse peptide sequences is essential for characterizing the thermodynamics of membrane stability. While structurally varied, AMPs primarily exert their lethal effects through physical permeabilization, making permeation studies vital for rational drug design and permeability forecasting [35]. In this study, PMF calculations were used to quantify the free energy of transfer from the aqueous phase into the bilayer, providing a thermodynamic basis for the observed membrane compromise and the energy required for this disruption. The translocation of the peptide into the bilayer interior is marked by an elevation in free energy, primarily driven by the loss of favorable electrostatic interactions with the lipid headgroups [93]. This high energy barrier suggests that while the peptide can effectively rupture the membrane to cause the cell permeabilization observed in SYTOX Green assays, the total passage of the peptide into the cytoplasm is an energetically demanding process.

While the present study comprehensively establishes the bactericidal efficacy of the liberated C-serum peptides, the mechanistic insights gained from the MD simulations require further experimental verification in complex biological systems. Although CSPH-AMP acts as a membrane-disruptive agent, a function supported by the proposed toroidal pore model, the computational model is inherently simplified, notably omitting complex bacterial cell wall structures. Furthermore, because the simulation evaluated only a single peptide molecule, it cannot account for ensemble-driven mechanisms of membrane disruption. Alternative mechanisms such as peptide aggregation, surfactant-like action, the carpet model, and barrel-stave pore formation rely on reaching a critical localized saturation threshold to achieve membranolysis, a dynamic that cannot be captured by single-molecule simulations [98]. The MD simulations provide molecular evidence for the spontaneous insertion of CSPH-AMP into the *S. aureus* lipid bilayer. This in silico biophysical disruption is directly corroborated by our in vitro study. The significant uptake of SYTOX Green confirms that the peptide-induced membrane strain observed in MD translates biologically into the formation of pores large enough to permit the influx of impermeable nucleic acid dyes. Furthermore, the macroscopic consequences of this destabilization are visually validated by SEM micrography, which illustrates severe morphological deformation, membrane wrinkling, and ultimate cellular lysis following CSPH-AMP exposure [99]. While the correlation between MD bilayer penetration, SYTOX Green pore formation, and SEM morphological destruction establishes a clear membrane-targeting mechanism, further biophysical assays are required to map the intermediate kinetic steps of cell death. Future research will prioritize membrane depolarization assays, utilizing voltage-sensitive dyes such as DiSC3(5), to capture the immediate collapse of the transmembrane electrical potential that theoretically precedes the large-scale pore formation detected by SYTOX Green [100]. Additionally, biomimetic liposome leakage assays (e.g., calcein efflux) utilizing synthetic vesicles matched exactly to our in silico DPPG/Lysyl-DPPG lipid ratios will be conducted [101]. This will isolate the lipid-peptide interaction from the complexities of the live staphylococcal cell wall, bridging the computational reaction coordinates with in vitro thermodynamic reality. Furthermore, high-resolution Transmission Electron Microscopy (TEM) will be employed to visualize the cross-sectional intracellular voiding and membrane detachment, complementing the surface topography captured by SEM. Furthermore, the transient nature of toroidal pores or localized membrane disruption often allows AMPs to translocate to the inner cytoplasmic leaflet and enter the cytoplasm. Consequently, further investigations are required to determine whether CSPH-AMP exhibits a multi-hit mechanism of action by targeting intracellular components following initial membrane permeabilization. Once internalized, such AMPs are known to block critical cellular processes by binding to DNA or RNA, or by interfering with protein synthesis [102]. This potential for intracellular translocation and subsequent disruption of enzymatic activity provides a strong theoretical basis for the widespread systemic stress and differential protein expression observed in our proteomic (LFQ) and PPI network analyses. Validating these specific mechanisms of action represents a critical avenue for our future research. Subsequent studies will prioritize membrane depolarization assays to map the kinetics of pore formation and differentiate between the carpet and toroidal models. Additionally, direct binding assays to evaluate the interaction of CSPH-AMP with intracellular targets, such as nucleic acids or specific precursor molecules like lipid II [103], will be necessary to confirm whether this ultrashort peptide achieves bacterial clearance strictly through direct membrane lysis or via a synergistic cascade of intracellular metabolic disruption. Ultimately, the therapeutic efficacy of CSPH-AMP must be verified using animal infection models. These future studies will assess its pharmacokinetic and pharmacodynamic profiles, alongside rigorous toxicity assessments, to establish the peptide’s druggability and readiness for clinical trials [104].

Additionally, the transition of these novel AMPs into clinical therapeutics necessitates rigorous safety evaluations. Sequence analysis using the APD6 database revealed that CSPH-AMP (LLKKKY) shares its highest sequence similarity (62.5%) with L4K3W^4^ (LLKWLKKL) and L3K3W^4^ (LLKWLKK), which are both de novo designed peptides. Previous studies have demonstrated that at 200 μg/mL, these peptides exhibit less than 5% hemolytic activity relative to a 0.2% Triton X-100 positive control [63]. Although this structural homology suggests a potentially favorable safety profile for CSPH-AMP, direct experimental validation remains mandatory. Because AMPs exert their effects via physical membrane permeabilization, there is an inherent risk of off-target cytotoxicity against mammalian host cells [105]. A limitation of the current work is the absence of preliminary in vitro safety profiling, such as hemolysis and cytotoxicity assays. Therefore, future investigations must prioritize establishing the therapeutic index of CSPH-AMP by determining its half-maximal hemolytic concentration (HC_50_) and half-maximal cytotoxic concentration (CC_50_) relative to its MIC against standard mammalian cell lines. Furthermore, comprehensive in vivo toxicity studies, including median lethal dose (LD_50_) assessments, will be crucial to define the lowest observed effect level (LOEL) and no observed effect level (NOEL). Addressing these safety parameters is an essential next step in validating the viability of upcycled rubber-latex peptides as safe and effective clinical antibiotics. The clinical translation of many traditional antimicrobial peptides is frequently limited by factors such as in vivo instability, potential host toxicity, and high manufacturing costs. Because approximately 88% of documented AMPs range from 11 to 50 amino acids in length, large-scale chemical synthesis can be economically prohibitive. The identified candidate, CSPH-AMP (LLKKKY), consists of six residues, classifying it as an ultrashort AMP (USAMP) [106]. USAMPs, typically defined as 3 to 8 amino acids in length, have been proposed as a strategy to mitigate these translational barriers, as their reduced length corresponds to lower synthesis costs and potentially enhanced stability in vivo [107]. The efficacy of USAMPs depends on a specific balance between positive charge density and hydrophobicity. Previous studies indicate that a localized positive charge is necessary to facilitate electrostatic accumulation at the negatively charged bacterial membrane, while hydrophobic residues are required to anchor the peptide and facilitate membrane insertion. Specifically, short peptides (5 to 9 residues) possessing a net charge of +3 to +5 and containing leucine and tryptophan residues have demonstrated inhibitory activity against MRSA, with minimum inhibitory concentrations (MICs) ranging from 3.125 to 6.25 μg/mL [108]. The primary sequence of CSPH-AMP incorporates this necessary structural balance; the cationic lysine constituents provide a theoretical basis for initial electrostatic attraction, while the hydrophobic residues offer the potential for membrane interaction. This physicochemical profile aligns with the structural requirements commonly observed in active hexapeptides USAMPs and three lysine residue lipopeptides [109,110]. Furthermore, the identification of CSPH-AMP highlights an alternative approach to AMP discovery pipelines. Currently, nearly 90% of the USAMPs documented in established repositories, such as the DBAASP database, are synthetically designed [111]. The identification of a USAMP with favorable physicochemical properties directly from a complex protein hydrolysate suggests that unexploited protein byproducts can serve as a viable source for bioactive peptides. This approach represents a resource-efficient methodology for identifying novel therapeutic templates, offering an alternative to traditional synthetic design or the screening of natural biological secretions [112].

## 5. Conclusions

A novel antimicrobial peptide, designated CSPH-AMP, was successfully identified and characterized following the enzymatic hydrolysis of residual proteins extracted from latex C-serum waste. These findings demonstrate that this peptide exhibits potent bactericidal activity against MRSA while maintaining robust stability. The in vitro assays and in silico MD simulations confirm that CSPH-AMP operates by destabilizing the bacterial lipid bilayer, resulting in membrane leakage and rapid cell death. Future investigations will focus on the in vivo efficacy and safety profile of this peptide, alongside rigorous pharmaceutics research to optimize its stability and delivery mechanisms for clinical application.

## Figures and Tables

**Table 1:** The purification balance sheet of antibacterial substance from hydrolyzed *H. brasiliensis* latex wastewater.

Purification Step	Total Volume (mL)	Total Dried Weight (mg)	Arbitrary Activity (AU/mL)	Total Activity (AU)	Specific Activity (AU/mg)	Purification Factor (Folds)	Yield (%)
Crude hydrolysate of protein in C-serum	1020.42	2591.18	20	20,408.40	7.88	1.00	100
Cation exchange chromatography	79.16	37.27	80	6332.80	169.92	21.57	31.03
Reversed-phase chromatography	9.64	1.59	160	1542.40	970.06	123.17	7.56

**Table 2:** The MIC, MBC, MBIC, and MBEC values of CSPH-AMP and standard antibiotics (vancomycin and cefoxitin) against susceptible and methicillin-resistant *S. aureus* strains (the reported values represent the statistical mode across the replicates; *n* = 3).

Tested Samples		*S. aureus* TISTR 517	MRSA Strain 142	MRSA Strain 1096	MRSA Strain 2468
Purified fraction of CSPH-AMP and synthesized peptide	MIC (μg/mL)	4	4	4	4
	MBC (μg/mL)	16	16	16	16
	MBIC (μg/mL)	16	16	16	16
	MBEC (μg/mL)	128	128	128	128
Vancomycin	MIC (μg/mL)	2	2	2	2
	MBC (μg/mL)	2	2	2	2
	MBIC (μg/mL)	8	8	8	8
	MBEC (μg/mL)	64	64	64	64
Cefoxitin	MIC (μg/mL)	2	>128	>128	>128
	MBC (μg/mL)	2	>128	>128	>128
	MBIC (μg/mL)	4	>128	>128	>128
	MBEC (μg/mL)	32	>128	>128	>128

**Table 3:** Residual antimicrobial activity of CSPH-AMP against MRSA strain 2468. The stability of the AMP was tested under varying conditions of temperature, pH, proteolytic digestion, and surfactant exposure.

Treatment Conditions	Residual Activity (%) of CSPH-AMP Against MRSA Strain 2468 (Mean ± SD; *n* = 3)			
Effect of temperature variation	1 h	6 h		12 h
40 °C	100.36 ± 1.65	99.46 ± 2.16		100.91 ± 0.31
60 °C	99.82 ± 0.83	98.38 ± 1.95		101.45 ± 1.26
80 °C	100.18 ± 0.83	100.36 ± 0.82		99.82 ± 2.06
100 °C	100.90 ± 2.98	99.46 ± 0.93		100.36 ± 2.69
Non-treatment (4 °C)	100.00 ± 1.25	100.00 ± 2.04		100.00 ± 1.37
Effect of autoclave condition	15 min		30 min	
121 °C and 15 psi	93.68 ± 1.25 *		88.81 ± 2.25 *^†^	
Non-treatment	100.00 ± 0.31			
Effect of protease digestion	1 h	6 h		12 h
CSPH-AMP with proteinase K (1 mg/mL)	98.55 ± 2.46	94.42 ± 3.06 *		85.48 ± 2.34 *^‡^
CSPH-AMP with trypsin (1 mg/mL)	99.46 ± 1.09	94.24 ± 1.08 *^†^		86.20 ± 1.73 *^‡^
CSPH-AMP with α-chymotrypsin (1 mg/mL)	97.64 ± 0.83	93.53 ± 0.31 *		85.30 ± 1.64 *^‡^
Non-treatment	100.00 ± 1.57	100.00 ± 1.12		100.00 ± 1.08
Effect of surfactant	1 h	6 h		12 h
CSPH-AMP with CTAB (1% *w*/*v*)	100.00 ± 1.42	99.29 ± 2.94		101.62 ± 1.08
CSPH-AMP with SDS (1% *w*/*v*)	108.05 ± 2.84 *	108.01 ± 2.45 *		109.34 ± 1.65 *
CSPH-AMP with Triton X-100 (1% *w*/*v*)	122.18 ± 1.88 *^§^	123.31 ± 4.31 *^§^		125.67 ± 3.67 *^§^
CTAB (1% *w*/*v*) alone	98.39 ± 1.42	99.82 ± 2.22		102.15 ± 1.94
SDS (1% *w*/*v*) alone	104.29 ± 1.94 *	103.56 ± 1.34 *		107.90 ± 1.12 *
Triton X-100 (1% *w*/*v*) alone	0.00 ± 0.00 *	0.00 ± 0.00 *		0.00 ± 0.00 *
Non-treatment	100.00 ± 0.82	100.00 ± 1.54		100.00 ± 2.24
Effect of pH variation	1 h	6 h		12 h
pH 1.2	153.05 ± 2.04 *	150.45 ± 1.65 *		153.75 ± 1.45 *
pH 4.5	110.22 ± 1.94 *	109.69 ± 3.00 *		111.33 ± 1.68 *
pH 6.8	101.08 ± 1.08	99.46 ± 0.54		101.46 ± 2.22
pH 7.4	100.00 ± 0.93	99.10 ± 1.36		100.18 ± 0.84
Non-treatment	100.00 ± 1.42	100.00 ± 0.31		100.00 ± 1.27

* The statistical significance of activity retention was determined by comparing treatments to the control within each time point using a one-way ANOVA (*p* < 0.05). ^†^ The statistical significance of activity retention was determined by comparing the 6 h time point to the 1 h time point using a one-way ANOVA (*p* < 0.05). ^‡^ The statistical significance of activity retention was determined by comparing the 12 h time point to the 6 h time point using a one-way ANOVA (*p* < 0.05). ^§^ The statistical significance of activity retention was determined by comparing surfactant alone to surfactant with CSPH-AMP using a one-way ANOVA (*p* < 0.05).

## Data Availability

The original contributions presented in this study are included in the article/Supplementary Materials. Further inquiries can be directed to the corresponding author.

## Supplementary Materials

The following supporting information can be downloaded at https://www.mdpi.com/article/10.3390/life16101655/s1, Table S1: Identified proteins from the functional enrichment proteomics profile of *Staphylococcus aureus* following CSPH-AMP treatment.; Table S2: Gene Ontology (GO) biological process enrichment analysis of expressed proteins in *Staphylococcus aureus* TISTR 517 following CSPH-AMP treatment.; Table S3: Gene Ontology (GO) biological process enrichment analysis of expressed proteins in methicillin-resistant *Staphylococcus aureus* strain 2468 following CSPH-AMP treatment.; Video S1: Molecular dynamics simulation trajectory of CSPH-AMP interacting with the simulated *Staphylococcus aureus* lipid membrane.
