# Supplementary material for: Anti-MRSA Activity and Molecular Dynamics Simulations of a Novel Peptide from Hevea brasiliensis Latex Protein Waste Hydrolysates
Source: Life (Basel). 2026 Oct 1;16(10):1655. doi: 10.3390/life16101655 (PMC13705795; doi:10.3390/life16101655)
Supplement: Supplementary file 1 [file life-16-01655-s001.zip › Supplementary file.pdf]

Anti-MRSA Activity and Molecular Dynamics Simulations of a Novel Peptide from *Hevea brasiliensis* Latex Protein Waste Hydrolysates

Namfa Sermkaew [1,2], Apichart Atipairin [1,2], Sucheewin Krobthong [3], Chanat Aonbangkhen [3,4], Yodying Yingchutrakul [5], Jumpei Uchiyama [6] and Nuttapon Songnaka [1,2,*]

Table S1. Identified proteins from the functional enrichment proteomics profile of *Staphylococcus aureus* following CSPH-AMP treatment.

ABD29386.1	PTS system, glucose-specific IIBC component, putative.
ABD29430.1	Conserved hypothetical protein.
ABD29455.1	5'-nucleotidase, lipoprotein e(P4) family.
ABD29571.1	Conserved hypothetical protein.
ABD29572.1	Conserved hypothetical protein.
ABD29609.1	DNA polymerase III, delta prime subunit, putative.
ABD29641.1	Conserved hypothetical protein; Belongs to the cysteine synthase/cystathionine beta- synthase family.
ABD29781.1	Penicillin-binding protein 4, putative; Belongs to the peptidase S11 family.
ABD29793.1	Conserved hypothetical protein.
ABD29873.1	Conserved hypothetical protein.
ABD29981.1	Conserved hypothetical protein; Nitronate monooxygenase that uses molecular oxygen to catalyze the oxidative denitrification of alkyl nitronates. Acts on propionate 3-nitronate (P3N), the presumed physiological substrate. Probably functions in the detoxification of P3N, a metabolic poison produced by plants and fungi as a defense mechanism.
ABD30027.1	Signal peptidase IA, putative; Belongs to the peptidase S26 family.
ABD30163.1	Dihydrolipoamide dehydrogenase.
ABD30228.1	Fibrinogen-binding protein.
ABD30575.1	DNA-binding protein HU, putative; Histone-like DNA-binding protein which is capable of wrapping DNA to stabilize it, and thus to prevent its denaturation under extreme environmental conditions.
ABD30607.1	Phage tail tape meausure protein, TP901 family, core region domain protein.
ABD30629.1	Conserved hypothetical phage protein.
ABD30644.1	Phage encoded DNA polymerase I.
ABD30702.1	acetyl-CoA carboxylase, biotin carboxyl carrier protein; This protein is a component of the acetyl coenzyme A carboxylase complex; first, biotin carboxylase catalyzes the carboxylation of the carrier protein and then the transcarboxylase transfers the carboxyl group to form malonyl-CoA.
ABD30850.1	Conserved hypothetical protein.
ABD30958.1	Conserved hypothetical protein.
ABD30984.1	Conserved hypothetical protein.
ABD31021.1	Protoporphyrinogen oxidase; Catalyzes the 6-electron oxidation of protoporphyrinogen-IX to form protoporphyrin-IX.
ABD31089.1	Conserved hypothetical phage protein.
ABD31095.1	Phi Mu50B-like protein.

ABD31236.1	Prohead protease.
ABD31330.1	Conserved hypothetical protein; Belongs to the SprT family.
ABD31397.1	Conserved hypothetical protein.
ABD31579.1	Hypothetical protein.
ahpC	Alkyl hydroperoxide reductase; Thiol-specific peroxidase that catalyzes the reduction of hydrogen peroxide and organic hydroperoxides to water and alcohols, respectively. Plays a role in cell protection against oxidative stress by detoxifying peroxides. Is important for survival under desiccation conditions. Not required for virulence although is necessary for nasal colonization.
argF	Ornithine carbamoyltransferase; Reversibly catalyzes the transfer of the carbamoyl group from carbamoyl phosphate (CP) to the N(epsilon) atom of ornithine (ORN) to produce L-citrulline; Belongs to the aspartate/ornithine carbamoyltransferase superfamily. OTCase family.
argF-2	Ornithine carbamoyltransferase; Reversibly catalyzes the transfer of the carbamoyl group from carbamoyl phosphate (CP) to the N(epsilon) atom of ornithine (ORN) to produce L-citrulline; Belongs to the aspartate/ornithine carbamoyltransferase superfamily. OTCase family.
argH	Argininosuccinate lyase.
atl	Bifunctional autolysin precursor, putative; Endohydrolysis of the di-N-acetylchitobiosyl unit in high- mannose glycopeptides and glycoproteins containing the -[(Man)5(GlcNAc)2]-Asn structure. One N-acetyl-D-glucosamine residue remains attached to the protein; the rest of the oligosaccharide is released intact. Cleaves the peptidoglycan connecting the daughter cells at the end of the cell division cycle, resulting in the separation of the two newly divided cells. Acts as an autolysin in penicillin-induced lysis (By similarity); In the C-terminal section; belongs to the glycosyl hydrolas [...]
ecfT	Cobalt transport protein, putative; Transmembrane (T) component of an energy-coupling factor (ECF) ABC-transporter complex. Unlike classic ABC transporters this ECF transporter provides the energy necessary to transport a number of different substrates.
eno	Enolase; Catalyzes the reversible conversion of 2-phosphoglycerate into phosphoenolpyruvate. It is essential for the degradation of carbohydrates via glycolysis; Belongs to the enolase family.
fda	Fructose-bisphosphate aldolase class-I, putative.
frr	Ribosome recycling factor; Responsible for the release of ribosomes from messenger RNA at the termination of protein biosynthesis. May increase the efficiency of translation by recycling ribosomes from one round of translation to another; Belongs to the RRF family.
ftnA	Ferritin, putative; Iron-storage protein; Belongs to the ferritin family. Prokaryotic subfamily.
gcvPB	Glycine cleavage system P-protein subunit ll, putative; The glycine cleavage system catalyzes the degradation of glycine. The P protein binds the alpha-amino group of glycine through its pyridoxal phosphate cofactor; CO(2) is released and the remaining methylamine moiety is then transferred to the lipoamide cofactor of the H protein; Belongs to the GcvP family. C-terminal subunit subfamily.
greA	Transcription elongation factor GreA; Necessary for efficient RNA polymerase transcription elongation past template-encoded arresting sites. The arresting sites in DNA have the property of trapping a certain fraction of elongating RNA polymerases that pass through, resulting in locked ternary complexes. Cleavage of the nascent transcript by cleavage factors such as GreA or GreB allows the resumption of elongation from the new 3'terminus. GreA releases sequences of 2 to 3 nucleotides.
groL	Chaperonin, 60 kDa, GrpEL, putative; Prevents misfolding and promotes the refolding and proper assembly of unfolded polypeptides generated under stress conditions.

infB	Translation initiation factor IF-2; One of the essential components for the initiation of protein synthesis. Protects formylmethionyl-tRNA from spontaneous hydrolysis and promotes its binding to the 30S ribosomal subunits. Also involved in the hydrolysis of GTP during the formation of the 70S ribosomal complex; Belongs to the TRAFAC class translation factor GTPase superfamily. Classic translation factor GTPase family. IF-2 subfamily.
lacB	Galactose-6-phosphate isomerase, LacB subunit.
lacG	6-phospho-beta-galactosidase; Belongs to the glycosyl hydrolase 1 family.
ldh2	L-lactate dehydrogenase; Catalyzes the conversion of lactate to pyruvate (Potential). Contributes to S.aureus growth during nitrosative stress in both aerobically and anaerobically cultured cells, despite playing a secondary role in this resistance mechanism (By similarity).
ligA	DNA ligase, NAD-dependent; DNA ligase that catalyzes the formation of phosphodiester linkages between 5'-phosphoryl and 3'-hydroxyl groups in double- stranded DNA using NAD as a coenzyme and as the energy source for the reaction. It is essential for DNA replication and repair of damaged DNA.
moaA	Molybdopterin cofactor biosynthesis protein A, putative; Catalyzes the cyclization of GTP to (8S)-3',8-cyclo-7,8-dihydroguanosine 5'-triphosphate.
nth	Endonuclease III, putative; DNA repair enzyme that has both DNA N-glycosylase activity and AP-lyase activity. The DNA N-glycosylase activity releases various damaged pyrimidines from DNA by cleaving the N-glycosidic bond, leaving an AP (apurinic/apyrimidinic) site. The AP-lyase activity cleaves the phosphodiester bond 3' to the AP site by a beta-elimination, leaving a 3'-terminal unsaturated sugar and a product with a terminal 5'-phosphate.
pflB	Formate acetyltransferase; Belongs to the glycyl radical enzyme (GRE) family. PFL subfamily.
pnp	Polyribonucleotide nucleotidyltransferase, putative; Involved in mRNA degradation. Catalyzes the phosphorolysis of single-stranded polyribonucleotides processively in the 3'- to 5'- direction.
prfB	Peptide chain release factor 2, putative; Peptide chain release factor 2 directs the termination of translation in response to the peptide chain termination codons UGA and UAA.
pyk	Pyruvate kinase; Belongs to the pyruvate kinase family.
pyrG	CTP synthase; Catalyzes the ATP-dependent amination of UTP to CTP with either L-glutamine or ammonia as the source of nitrogen. Regulates intracellular CTP levels through interactions with the four ribonucleotide triphosphates.
recA	Recombination protein RecA, putative; Can catalyze the hydrolysis of ATP in the presence of single- stranded DNA, the ATP-dependent uptake of single-stranded DNA by duplex DNA, and the ATP-dependent hybridization of homologous single-stranded DNAs. It interacts with LexA causing its activation and leading to its autocatalytic cleavage; Belongs to the RecA family.
rplC	Ribosomal protein L3, putative; One of the primary rRNA binding proteins, it binds directly near the 3'-end of the 23S rRNA, where it nucleates assembly of the 50S subunit; Belongs to the universal ribosomal protein uL3 family.
rplM	Ribosomal protein L13; This protein is one of the early assembly proteins of the 50S ribosomal subunit, although it is not seen to bind rRNA by itself. It is important during the early stages of 50S assembly.
rplQ	Ribosomal protein L17.
rplW	Ribosomal protein L23, putative; One of the early assembly proteins it binds 23S rRNA. One of the proteins that surrounds the polypeptide exit tunnel on the outside of the ribosome. Forms the main docking site for trigger factor binding to the ribosome; Belongs to the universal ribosomal protein uL23 family.
rpmD	Ribosomal protein L30.
rpoA	DNA-directed RNA polymerase alpha chain, putative; DNA-dependent RNA polymerase catalyzes the transcription of DNA into RNA using the four ribonucleoside triphosphates as substrates.
rpoB	RNA polymerase beta chain, putative; DNA-dependent RNA polymerase catalyzes the transcription of DNA into RNA using the four ribonucleoside triphosphates as substrates.
rpsC	Ribosomal protein S3; Binds the lower part of the 30S subunit head. Binds mRNA in the 70S ribosome, positioning it for translation; Belongs to the universal ribosomal protein uS3 family.
rpsD	Ribosomal protein S4; One of the primary rRNA binding proteins, it binds directly to 16S rRNA where it nucleates assembly of the body of the 30S subunit.
rpsF	Ribosomal protein S6; Binds together with S18 to 16S ribosomal RNA.

rpsH	Ribosomal protein S8, putative; One of the primary rRNA binding proteins, it binds directly to 16S rRNA central domain where it helps coordinate assembly of the platform of the 30S subunit; Belongs to the universal ribosomal protein uS8 family.
rpsK	Ribosomal protein S11, putative; Located on the platform of the 30S subunit, it bridges several disparate RNA helices of the 16S rRNA. Forms part of the Shine- Dalgarno cleft in the 70S ribosome; Belongs to the universal ribosomal protein uS11 family.
rpsL	Ribosomal protein S12; With S4 and S5 plays an important role in translational accuracy.
rpsN	Ribosomal protein S14p/S29e; Binds 16S rRNA, required for the assembly of 30S particles and may also be responsible for determining the conformation of the 16S rRNA at the A site; Belongs to the universal ribosomal protein uS14 family.
rpsO	Ribosomal protein S15; One of the primary rRNA binding proteins, it binds directly to 16S rRNA where it helps nucleate assembly of the platform of the 30S subunit by binding and bridging several RNA helices of the 16S rRNA.
rpsS	Ribosomal protein S19; Protein S19 forms a complex with S13 that binds strongly to the 16S ribosomal RNA.
rpsZ	Ribosomal protein S14p/S29e, putative; Binds 16S rRNA, required for the assembly of 30S particles and may also be responsible for determining the conformation of the 16S rRNA at the A site.
rsgA	Conserved hypothetical protein; One of several proteins that assist in the late maturation steps of the functional core of the 30S ribosomal subunit. Helps release RbfA from mature subunits. May play a role in the assembly of ribosomal proteins into the subunit. Circularly permuted GTPase that catalyzes slow GTP hydrolysis, GTPase activity is stimulated by the 30S ribosomal subunit; Belongs to the TRAFAC class YlqF/YawG GTPase family. RsgA subfamily.
sarR	Conserved hypothetical protein; Negative regulator of sarA transcription at late exponential and stationary growth phases. It contributes to the modulation of target genes downstream of the sarA regulatory cascade. Also, positively regulates expression of primary transcripts RNAII and RNAIII generated by agr (virulence accessory gene regulator) locus.
sasG	Conserved hypothetical protein; Promotes adhesion of bacterial cells to human squamous nasal epithelial cells, a phenomenon which is likely to be important in nasal colonization. Forms short, extremely dense and thin fibrils all over the bacterial surface. Does not bind to either buccal cells or non- differentiated keratinocytes. Promotes cellular aggregation leading to biofilm formation.
sl11	Conserved hypothetical protein; Secreted protein that plays a role in the inhibition of host immune system. Targets myeloid cells such as monocytes or granulocytes through binding with sialyllactosamine-containing glycoproteins. Prevents initial rolling of neutrophils toward the site of infection by interacting with host SELPLG. Disrupts neutrophil motility by induction of cell adhesion via interacting with glycans but independently of SELPLG; Belongs to the staphylococcal/streptococcal toxin family.
sodA	Superoxide dismutase, Mn, putative; Destroys superoxide anion radicals which are normally produced within the cells and which are toxic to biological systems. May play a role in maintaining cell viability throughout all stages of growth, but may be the major SOD activity in the exponential growth- phase. Has a role in resisting external superoxide stress. Involved in acid tolerance and the acid-adaptive response. Mediates the derepression of perR regulon in the response to HOCl stress when the level of SOD activity is low.
tilS	Conserved hypothetical protein; Ligates lysine onto the cytidine present at position 34 of the AUA codon-specific tRNA(Ile) that contains the anticodon CAU, in an ATP-dependent manner. Cytidine is converted to lysidine, thus changing the amino acid specificity of the tRNA from methionine to isoleucine.
truA	tRNA pseudouridine synthase A; Formation of pseudouridine at positions 38, 39 and 40 in the anticodon stem and loop of transfer RNAs.
trxA	Thioredoxin; Component of the thioredoxin-thioredoxin reductase system. Participates in various redox reactions through the reversible oxidation of its active center dithiol to a disulfide and catalyzes dithiol-disulfide exchange reactions (By similarity).
tuf	Translation elongation factor Tu; This protein promotes the GTP-dependent binding of aminoacyl- tRNA to the A-site of ribosomes during protein biosynthesis.

Table S2. Gene Ontology (GO) biological process enrichment analysis of expressed proteins in *Staphylococcus aureus* TISTR 517 following CSPH-AMP treatment.

Biological Process	Strength	Signal	FDR*	Matching proteins in network
Glycolytic process	1.21	0.49	0.0252	eno,pyk,ABD31397.1,ldh2,fda
Carbohydrate catabolic process	0.9	0.45	0.0252	eno,pyk,ABD31397.1,lacB,ldh2,fda
Generation of precursor metabolites and energy	0.76	0.38	0.0386	eno,pyk,ABD31397.1,lacB,ldh2,fda
Translation	0.69	0.4	0.0252	rpsF,frr,rpsO,rpsN,rpsD,rplQ,rpsH,rpsZ,rplW,rplC
Peptide metabolic process	0.66	0.39	0.0252	rpsF,ABD30027.1,frr,rpsO,rpsN,rpsD,rplQ,rpsH,rpsZ,rplW,rplC
Cellular macromolecule biosynthetic process	0.52	0.35	0.0259	rpsF,ABD29781.1,frr,rpsO,rpsN,rpsD,rplQ,rpsH,rpsZ,rplW,rplC
Macromolecule biosynthetic process	0.51	0.35	0.0252	rpsF,ABD29609.1,ABD29781.1,frr,rpsO,rpsN,ABD30644.1,rpsD,rplQ,rpsH,rpsZ,rplW,rplC
Protein metabolic process	0.5	0.35	0.0252	rpsF,ABD29781.1,ABD30027.1,frr,rpsO,rpsN,rpsD,ABD31236.1,rplQ,rpsH,rpsZ,rplW,rplC,moaA
Gene expression	0.48	0.33	0.0325	rpsF,tilS,ABD30027.1,frr,rpsO,rpsN,rpsD,rplQ,rpsH,rpsZ,rplW,rplC
Organonitrogen compound metabolic process	0.37	0.33	0.0174	pflB,rpsF,ABD29641.1,ABD29781.1,eno,ABD30027.1,atl,argF,frr,rpsO,rpsN,gcvPB,pyk,rpsD,ABD31021.1,ABD31236.1,ABD31397.1,rplQ,rpsH,rpsZ,rplW,rplC,moaA,ldh2,fda,argF-2
Nitrogen compound metabolic process	0.27	0.28	0.0252	pflB,rpsF,ABD29609.1,tilS,ABD29641.1,ABD29781.1,eno,ABD30027.1,atl,argF,frr,rpsO,rpsN,ABD30644.1,gcvPB,pyk,rpsD,ABD31021.1,ligA,ABD31236.1,ABD31397.1,rplQ,rpsH,rpsZ,rplW,rplC,moaA,ldh2,fda,argF-2
Primary metabolic process	0.26	0.28	0.0252	pflB,rpsF,ABD29609.1,tilS,ABD29641.1,ABD29781.1,eno,ABD30027.1,argF,frr,rpsO,rpsN,ABD30644.1,ABD30702.1,gcvPB,pyk,rpsD,ligA,ABD31236.1,ABD31397.1,lacB,rplQ,rpsH,rpsZ,rplW,rplC,moaA,ldh2,fda,argF-2
Cellular metabolic process	0.25	0.27	0.0252	pflB,ABD29386.1,rpsF,ABD29609.1,tilS,ABD29641.1,ABD29781.1,eno,ABD30027.1,argF,frr,rpsO,rpsN,ABD30644.1,ABD30702.1,gcvPB,sodA,pyk,rpsD,ABD31021.1,ligA,ABD31397.1,lacB,rplQ,rpsH,rpsZ,rplW,rplC,moaA,ldh2,fda,argF-2
Metabolic process	0.22	0.26	0.0252	pflB,ABD29386.1,rpsF,ABD29609.1,tilS,ABD29641.1,ABD29781.1,eno,ABD30027.1,atl,argF,frr,rpsO,rpsN,ABD30644.1,ABD30702.1,gcvPB,sodA,pyk,rpsD,ABD31021.1,ligA,ABD31236.1,ABD31397.1,lacB,rplQ,rpsH,rpsZ,rplW,rplC,moaA,ldh2,fda,argF-2
Organic substance metabolic process	0.22	0.26	0.0267	pflB,rpsF,ABD29609.1,tilS,ABD29641.1,ABD29781.1,eno,ABD30027.1,atl,argF,frr,rpsO,rpsN,ABD30644.1,ABD30702.1,gcvPB,pyk,rpsD,ABD31021.1,ligA,ABD31236.1,ABD31397.1,lacB,rplQ,rpsH,rpsZ,rplW,rplC,moaA,ldh2,fda,argF-2
Cellular process	0.18	0.25	0.0252	pflB,ABD29386.1,rpsF,ABD29609.1,tilS,ABD29641.1,ABD29781.1,eno,ABD30027.1,atl,ABD30163.1,argF,rsgA,frr,rpsO,rpsN,ABD30575.1,ABD30644.1,ABD30702.1,gcvPB,sodA,pyk,rpsD,ABD31021.1,ftnA,ligA,groL,ABD31330.1,ABD31397.1,lacB,rplQ,rpsH,rpsZ,rplW,rplC,moaA,sasG,ldh2,fda,argF-2

* FDR: False Discovery Rate. Statistical significance was defined at $p < 0.05$. Protein identification and functional enrichment were performed by searching the experimental dataset against the *Staphylococcus aureus* (strain NCTC 8325) UniProt reference proteome (Proteome ID: UP000008816; Taxon ID: 93061; containing 2889 protein sequences).

Table S3. Gene Ontology (GO) biological process enrichment analysis of expressed proteins in methicillin-resistant *Staphylococcus* aureus strain 2468 following CSPH-AMP treatment.

Biological Process	Strength	Signal	FDR*	Matching proteins in network
Ribosomal small subunit biogenesis	1.57	0.81	0.0068	rsgA,rpsK,rpsS
Regulation of translation	1.4	1.18	0.00025	tuf,prfB,infB,rpsD,rplM
Transcription, DNA-templated	1.27	0.55	0.0307	rpoB,greA,rpoA
Translation	1.04	1.27	4.78E-07	rpsL,tuf,prfB,infB,rpsO,rpsD,rplM,rpsK,rpmD,rpsC,rpsS
Gene expression	0.93	1.27	9.81E-10	tilS,rpoB,rpsL,tuf,prfB,infB,rpsO,pnp,greA,rpsD,rplM,truA,rpoA,rpsK,rpmD,rpsC,rpsS
Macromolecule biosynthetic process	0.85	1.00	3.63E-07	rpoB,rpsL,tuf,prfB,infB,rpsO,greA,rpsD,rplM,rpoA,rpsK,rpmD,rpsC,rpsS
RNA metabolic process	0.77	0.53	0.0076	tilS,rpoB,pnp,greA,truA,rpoA,rpsK
Cellular nitrogen compound biosynthetic process	0.70	0.75	4.07E-06	rpoB,rpsL,tuf,prfB,infB,rpsO,greA,rpsD,pyrG,rplM,rpoA,rpsK,rpmD,rpsC,rpsS
Cellular macromolecule metabolic process	0.68	0.71	7.09E-06	rpsL,tuf,prfB,infB,rpsO,pnp,recA,nth,rpsD,ligA,rplM,rpsK,rpmD,rpsC,rpsS
Regulation of gene expression	0.66	0.39	0.0299	tuf,prfB,infB,pnp,greA,rpsD,rplM
Nucleic acid metabolic process	0.62	0.48	0.0043	tilS,rpoB,pnp,recA,nth,greA,ligA,truA,rpoA,rpsK
Macromolecule metabolic process	0.61	0.69	2.56E-07	tilS,rpoB,rpsL,tuf,prfB,infB,rpsO,pnp,recA,nth,greA,rpsD,ligA,rplM,truA,rpoA,rpsK,rpmD,rpsC,rpsS
Regulation of cellular process	0.61	0.39	0.0239	ahpC,tuf,prfB,trxA,infB,greA,rpsD,rplM
Cellular nitrogen compound metabolic process	0.6	0.69	4.79E-08	tilS,rpoB,rpsL,tuf,prfB,eno,infB,rpsO,pnp,recA,nth,greA,rpsD,ligA,pyrG,rplM,truA,rpoA,rpsK,rpmD,rpsC,rpsS
Regulation of biological process	0.59	0.40	0.0162	ahpC,tuf,prfB,trxA,infB,pnp,greA,rpsD,rplM
Organonitrogen compound biosynthetic process	0.55	0.48	0.0012	rpsL,tuf,prfB,argH,infB,rpsO,rpsD,pyrG,rplM,rpsK,rpmD,rpsC,rpsS
Cellular biosynthetic process	0.51	0.49	0.00029	rpoB,rpsL,tuf,prfB,argH,infB,rpsO,greA,rpsD,pyrG,rplM,rpoA,rpsK,rpmD,rpsC,rpsS
Nucleobase-containing compound metabolic process	0.51	0.40	0.0068	tilS,rpoB,eno,pnp,recA,nth,greA,ligA,pyrG,truA,rpoA,rpsK
Organic substance biosynthetic process	0.50	0.47	0.00036	rpoB,rpsL,tuf,prfB,argH,infB,rpsO,greA,rpsD,pyrG,rplM,rpoA,rpsK,rpmD,rpsC,rpsS
Primary metabolic process	0.49	0.57	4.79E-08	pflB,tilS,rpoB,rpsL,tuf,prfB,eno,argH,infB,rpsO,pnp,recA,nth,greA,rpsD,ligA,pyrG,lacG,rplM,truA,rpoA,rpsK,rpmD,rpsC,rpsS
Nitrogen compound metabolic process	0.48	0.55	2.52E-07	pflB,tilS,rpoB,rpsL,tuf,prfB,eno,argH,infB,rpsO,pnp,recA,nth,greA,rpsD,ligA,pyrG,rplM,truA,rpoA,rpsK,rpmD,rpsC,rpsS
Cellular metabolic process	0.45	0.51	2.56E-07	pflB,tilS,rpoB,rpsL,tuf,prfB,eno,argH,infB,rpsO,pnp,recA,nth,greA,rpsD,ligA,pyrG,lacG,rplM,truA,rpoA,rpsK,rpmD,rpsC,rpsS
Organonitrogen compound metabolic process	0.44	0.38	0.0043	pflB,rpsL,tuf,prfB,eno,argH,infB,rpsO,rpsD,pyrG,rplM,rpsK,rpmD,rpsC,rpsS
Organic substance metabolic process	0.42	0.48	5.89E-07	pflB,tilS,rpoB,rpsL,tuf,prfB,eno,argH,infB,rpsO,pnp,recA,nth,greA,rpsD,ligA,pyrG,lacG,rplM,truA,rpoA,rpsK,rpmD,rpsC,rpsS
Cellular process	0.35	0.43	4.79E-08	pflB,ahpC,tilS,rpoB,rpsL,tuf,prfB,eno,argH,trxA,rsgA,infB,rpsO,pnp,recA,nth,greA,rpsD,ligA,pyrG,lacG,rplM,truA,ecfT,rpoA,rpsK,rpmD,rpsC,rpsS

* FDR: False Discovery Rate. Statistical significance was defined at $p < 0.05$. Protein identification and functional enrichment were performed by searching the experimental dataset against the *Staphylococcus aureus* (strain NCTC 8325) UniProt reference proteome (Proteome ID: UP000008816; Taxon ID: 93061; containing 2889 protein sequences).
